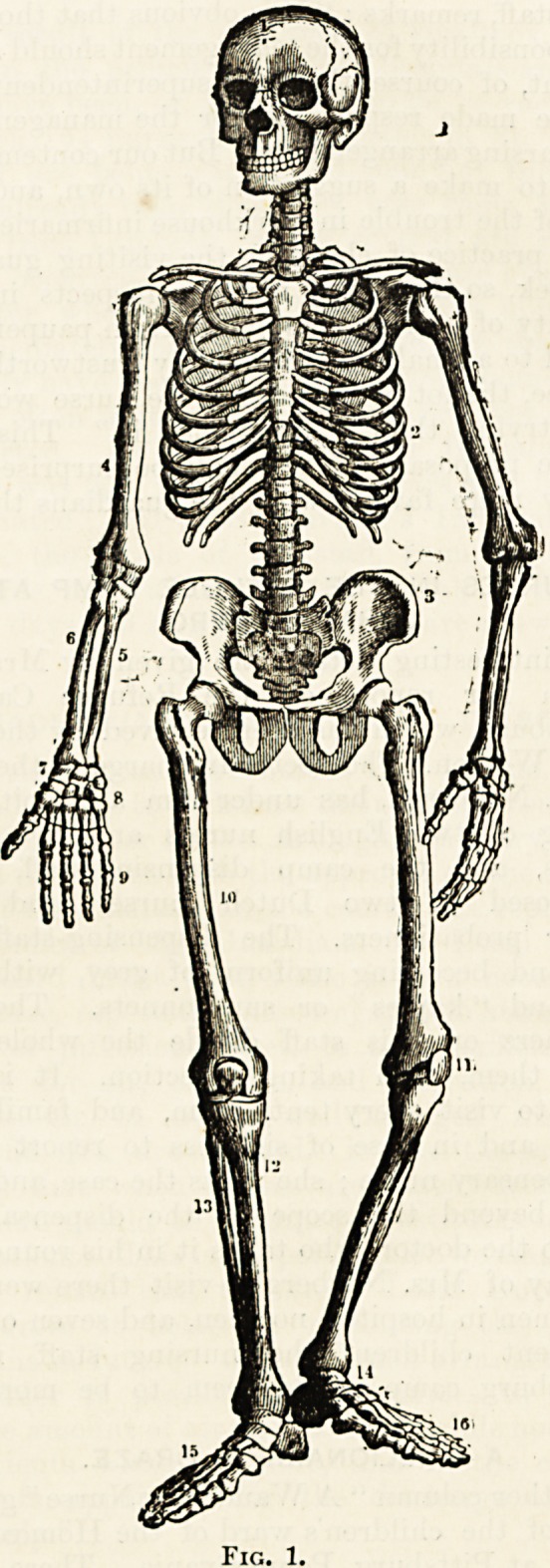# The Hospital. Nursing Section

**Published:** 1901-10-05

**Authors:** 


### The Hospital.

### Nursing Section.

Contributions for this Section of "The Hospital" should be addressed to the Editor, "The Hospital" Nursing Section, 28 & 29 Southampton Street, Strand, London, W.C.

No. 784.—Vol. XXXI. *SATURDAY, OCTOBER* 5, 1901.

In order to meet the requirements of the Post Office, we have discontinued the use of the sub-title of "Nursing Mirror." To-day, and in future, that portion of THE HOSPITAL which deals with nursing topics will be known as the "Nursing Section." This is the only change, and it in no way affects the features of the paper.

### Notes on News from the Nursing World.

### QUEEN ALEXANDRA'S IMPERIAL MILITARY NURSING SERVICE.

The announcement that Queen Alexandra is to be President of the Army and Indian Nursing Service, under the scheme of reorganisation which has been drawn up by the committee appointed by the Secretary of State for War, will give profound satisfaction to nurses in all parts of England and her colonies. This is, in fact, the most interesting personal feature of the scheme, and it is also one of the most significant. The Queen, who has done so much to promote the nursing movement and who has its welfare so thoroughly at heart, would neither have consented to be placed at the head of the Army Nursing Service nor allowed it to bear her name, unless she had assured herself that the provisions which the committee have drawn up would be for the benefit of those who enter the service, no less than for the advantage of the community. Elsewhere we give the whole of the details of the scheme, respecting which some of our readers may like to express their views; and all will be glad of the opportunity of studying it. The inclusion, in addition to the Royal President, of the Matron-in-Chief of Queen Alexandra's Imperial Military Nursing Service—who is also to be a member of the Advisory Board of the Royal Army Medical Corps, for nursing service—and of three matrons of large civil hospitals with medical schools, on the Nursing Board, is a guarantee that the nursing staff will always be treated with every possible consideration. As the matron-in-chief is to be invested with very large powers, the allotment of this post is a matter of primary importance. We hope that every care will be taken to secure the services of a lady whose name commands both respect and enthusiasm in the nursing world. Her three colleagues should also be matrons whose varied and valuable experience, as well as their present position, justifies their nomination to a seat on the Nursing Board. We are glad to see that all members of the Army Nursing Reserve who have been in military employment during the war in South Africa are to be eligible for appointment in the newly constituted Service, "if recommended by the N ursing Board"—a very essential proviso. The pay and allowances are on a liberal scale, and the age of retirement has not been fixed in accordance with the ridiculous theory that nurses have done their best work at 40. It is not pretended that the scheme is absolutely perfect, but our first impression is highly favourable, and we warmly congratulate Mr. Brodrick, Sir Edward Ward, and their colleagues on the manner in which they have endeavoured to meet the necessities of the case.

### THE COMING CORONATION.

With the commencement of autumn the thoughts of nurses, like those of most people, will turn to the coming Coronation of our King and Queen. A member of the Royal National Pension Fund, who writes to us on the subject, states accurately enough, we are sure, that no class would more appreciate the sight of the Coronation procession than nurses. Nor, if, as our correspondent suggests, there are many who are prepared to pay a moderate sum for the privilege, should there be any serious difficulty in the way. With regard to the direct appeal which our correspondent makes to us, we invite our readers who would like to avail themselves of any arrangement to write to us, and when we know their views more fully we shall be glad to make inquiries with the object of ascertaining the possibility of carrying them out.

### THE RECIPIENTS OF THE ROYAL RED CROSS.

The coveted decoration of the Royal Red Cross has been bestowed by the King upon a number of superintendents and nursing sisters who were commended in the despatches of Lord Roberts during the period he was Commander-in-Chief in South Africa. The members of the Army Nursing Service who have thus been honoured are as follows:—Sups. Miss M. Thomas, Miss S. J. Browne, Miss E. A. Dowse, Miss S. E. Webb, Miss S. E. Oram, and Miss A. Garriock; nursing sister and acting sups. Miss L. W. Tulloh and Miss L. M. Stewart; nursing sisters Miss E. T. Noble, Miss A. S. Bond, Miss J. Hoadley, Miss M. G. Hill, Miss E. Nixon, New South Wales; Miss A. Bidsmead, South Australia; and Miss J. M. N. Williamson, New Zealand. The decoration has also been awarded to nursing sisters Miss J. E. Skillman, Miss A. B. Smith, Miss A. B. Trew, Miss E. H. Beecher, Miss A. Knaggs, Miss J. Southwell, Miss E. M. Anderson, Miss E. M. McCarthy, and Miss M. E. Greenham, of the Army Nursing Service Reserve. Other recipients are Miss A. M. MacDonnell, of the Irish Hospital; Beatrice Constance, Lady Chesham, Miss C. E. Nisbet, and Miss M. A. Fisher, of the Yeomanry Hospital; Miss M. Lloyd, of the Welsh Hospital; Sister E. C. Lawrence, of Princess Christian's Hospital; Sister A. W. Gill, of the Edinburgh Hospital; Nurse E. Pretty, of the Portland National Hospital; Miss J. Underwood, for services at Ladybrand; Miss E. Ludlow, for services at Ladysmith; Lady Sarah Wilson, Mother Superior Teresa, Miss Hill, and Miss Craufurd, for services at Mafeking; and the Hon. Mrs. Agnes Mary Goldmann, Mrs. Gurning, Mrs. Maasdorp, Mrs. Wilman, and Miss Cairnnan, for services not specified in the *Gazette.* This, of course, is not an exhaustive list. There are nurses still hard at work in South Africa whose claims to the Royal Red Cross Lord Kitchener is not in the least likely to overlook at the proper time.

### THE WAR NURSES.

We are glad to see that Nursing Sisters Mary Ann Cain, L. Vereledge, and J. Paget were discharged from hospital to duty for the week ending September 15. The following are on passage home in the *Bavaria,* due October 14:—Nursing Sisters C. Hamilton, E. H. Hordley, L. A. H. Seligmann, A. Lawrence, and A. McLeod. Sister Culverwell is in the same vessel in charge of the invalids. On board the *Britannia,* which is due at Southampton on October 18th, are Nursing Sisters M. Boyd-King, L. J. Attree, M. Steel, and P. Frater. On board the *Simla,* which arrived at Southampton last week, were Nursing Sisters A. T. Young, B. Roberts, A. M. Winder, J. B. B. Bell, M. Cross, S. M. Lippiatt, and A. M. Gutteridge. All these, with the exception of Miss Gutteridge, whose time has expired, return to South Africa.

### OUR CLOTHING DISTRIBUTION.

Although we have already reminded the kind friends who enable us to make a distribution of clothing at Christmas to the matrons of a number of hospitals and infirmaries that we are again relying upon their help, it may be worth while to emphasise the fact that the garments most in request for the use of the patients are woollen shirts for the men, flannel petticoats for the women, and socks for the children. Of course, any warm article is acceptable in winter, but we indicate some which are especially suitable with the hope of saving trouble to any of our readers who may be in doubt on the point. We may repeat for the benefit of those who may not have seen our former intimation that the name and address of the sender of each parcel should be enclosed in order that the gift may be acknowledged in our columns, and that all parcels should be addressed The Editor, The Hospital, 28 & 29 Southampton Street, Strand, London, W.C., marked "Clothing Distribution." They should reach us not later than Monday, December 16th.

### THE HEBREW WARDS AT THE LONDON HOSPITAL.

The Day of Atonement, or, as it is familiarly called, "Black Monday," was observed as usual in the Jewish wards of the London Hospital from the evening of Saturday, September 21st, to sunset on Sunday. Only one patient, "Sister Rothschild" told our representative, was well enough to fast at all; this was a surgical case, and abstinence from breakfast was all that was possible under the circumstances. "So religiously is this day observed that even the most lax will keep it if they possibly can." All the patients joined in the prayers said by the Rabbi. The following Saturday the Feast of Tabernacles was observed, and for several days previously "Sister" had been keeping the bunches of palm and bay fresh in a bath. These were given in turn to each patient early on the morning of the feast-day, and a prayer was said. They were then carried round the ward, palm and bay in one hand and lime in the other, while prayers were recited by the Rabbi, and all who were able joined in the procession. Some of the patients in this ward can only talk Yiddish, but the nurses say they soon get accustomed to their language, and can understand enough to make out what the patient wants to say. At the one end of the women's ward is a Passover cake, hung over the door to bring good luck.

### NURSES AND VACCINATION.

As a result of the outbreak of smallpox, all the nurses in the principal London hospitals and infirmaries have been, or are about to be, re-vaccinated. This week 164 nurses and wardmaids in St. Thomas' Hospital are being vaccinated, many of the sisters having already been done. A case came into the hospital from Lambeth, where several are reported to have occurred. Thanks to the astute observance of a porter, who suspected the patient's appearance and immediately took him apart, the risk that would inevitably have been run in the waiting-room—where patients often wait together for two or three hours—was averted. At the Westminster Hospital every one who was last vaccinated six years ago has been re-vaccinated; and at St. Saviour's Infirmary, East Dulwich, the whole of the staff, from the matron downwards, have undergone the ordeal, at intervals of a few days, 25 at a time. There are a few "bad" arms.

### ARMAGH GUARDIANS BROUGHT TO BOOK.

The Irish Local Government Board stand to their guns, and refuse to withdraw their animadversions on the medical officer at the Armagh Infirmary because they did not meet with the approval of the Armagh Board of Guardians. In the course of a reply to the remonstrance of the Guardians the Local Government Board insist that, while it is a necessity for every nurse, especially those engaged in attending on arduous or infectious cases, to have sufficient time regularly for air, exercise, and recreation, "as is arranged for in every well-managed institution where the sick are treated," it is at the same time essential that when nurses are absent from such a cause there should be qualified substitutes to attend to the patients. This is the point which we emphasised, and we rejoice that the Irish Local Government Board drive it home by requesting the Armagh Guardians in future "to make such arrangements as will render it possible for the nurses to take the requisite amount of air and exercise, while not leaving the patients without nurses, and in the charge of attendants appointed for the discharge of menial duties only."

### AN EXCELLENT EXAMPLE AT BOOTLE.

The committee of the Bootle Free Library have decided to grant for the use of the nursing staff at the Linacre Hospital a hundred standard works from the stock of duplicate books. As it is obviously impossible for the nurses of a hospital for infectious diseases to enjoy the advantages offered by a free library, a gift of this kind is a great boon, and we hope that other free library committees will see their way to be equally considerate. Private individuals might also, when they have a few readable books to spare, think of the nurses who, from the nature of their duties, cannot borrow.

### A PROPOSAL TO ABOLISH VISITING GUARDIANS.

An influential provincial contemporary, alluding to our contention that a better plan than the new departure of the Gainsborough Board of Guardians, in dispensing with the attendance of candidates for nurses' appointments before them, would be to invest the superintendent nurse with the authority to choose her own staff, remarks : "It is obvious that those who have responsibility for the management should select." We meant, of course, that the superintendent nurse should be made responsible for the management of all the nursing arrangements. But our contemporary goes on to make a suggestion of its own, and says : "Much of the trouble in workhouse infirmaries arises from the practice of changing the visiting guardians every week, so that every member inspects in turn. If the duty of hearing complaints from paupers were delegated to a small but thoroughly trustworthy sub-committee, the lot of the workhouse nurse would be far less trying than it frequently is." This is an admirable proposal, but we shall be surprised if it finds any more favour with the guardians than our own.

### NURSES IN THE REFUGEE CAMP AT JOHANNESBURG.

Some interesting details are given by Mrs. Neybergh in her report on the Refugee Camp at Johannesburg, which has been received by the Guild of Loyal Women. The doctor in charge of the camp, says Mrs. Neybergh, has under him a hospital staff consisting of two English nurses and five Dutch assistants, and the camp dispensing-staff, which is composed of two Dutch nurses and eight volunteer probationers. The dispensing-staff wear a neat and becoming uniform of grey, with white aprons and "kapjes" or sun-bonnets. The eight probationers on this staff divide the whole camp between them, each taking a section. It is their business to visit every tent, room, and family each morning, and in case of sickness to report to the head dispensary nurse ; she visits the case, and if she finds it beyond the scope of the dispensary she reports to the doctor, who takes it in his round. As on the day of Mrs. Neybergh's visit there were only eight women in hospital, no men, and seven or eight convalescent children, the nursing staff in the Johannesburg camp would seem to be more than adequate.

### A MILLIONAIRE'S CRAZE.

In another column "A Wandering Nurse" gives an account of the children's ward of the Homœopathic Hospital at Pittsburg, Pennsylvania. There is one notable omission from her description of this sumptuously furnished apartment. Nothing is said about the nurses in charge of the millionaire's "model ward." We should really like to know something about them. Sick children who can only eat with silver spoons and forks, repose in cots of white enamel with white and hand-embroidered bedspreads, perform their ablutions in crockery decorated with tropical birds, and fall asleep in perfumed sheets, must need the services of nurses appropriately attired. We can imagine them gliding about their duties in pale pink washing-silk frocks, with dainty lace-trimmed aprons and caps, both embroidered with pale-pink roses, the chains of their chatelaines formed by wreaths of rosebuds ; and equally, of course, the matron makes it an inflexible rule that no nurse who does not also carry roses on her cheek shall be admitted to the "bower of roses."

### SUDDEN DEATH OF A PROBATIONER.

The death of Miss Mary Eveline Radford, who had just finished her training at the Stockton-on-Tees Hospital, has occurred under singularly distressing circumstances. Prior to taking up new duties at Malvern, Miss Radford left Stockton on Monday last week to pay a visit to her former matron, Miss Bemrose, now matron of the Cottage Hospital, Ebbw Vale, and became seriously ill in the train. On her arrival at Ebbw Vale she was in a most critical condition. Medical assistance was at hand, and it was found that she was suffering from pneumonia. After patiently enduring terrible suffering, she quietly passed away on Thursday, to the grief of a numerous circle of friends.

### LITERATURE FOR INVALID SOLDIERS.

In reply to a correspondent who last week asked how literature could be sent out to South Africa for the use of invalid soldiers returning home, ex-Sergt. H. J. Johnson, 85 Wigmore Street, London, W., kindly informs us that to those who will write to him, he will send box labels and postal wrappers free of cost, which will ensure the parcels reaching the right quarters. In the case of postal packages the necessary stamps must, of course, be affixed ; but boxes will be forwarded free, if carriage is paid to Southampton.

### "WATCHED BY NURSES."

It is announced that Madame Auguste Christensen, of Copenhagen, is to attempt to totally abstain from food for thirty days, commencing on October 10th, at the Westminster Aquarium, and that "during the whole period she will be watched by nurses day and night, and be under medical supervision." We trust that no trained nurses have consented to have anything to do with the foolhardy person who, assuming that she has no idea of deception, proposes to run the risk of killing herself in order to gratify the morbid curiosity of a section of the public. The fact that Madame Christensen made an attempt of the same kind some years since, but was compelled to abandon it on the eighteenth day, ought to be a quite sufficient reason why she should not tempt Providence again. But in any case a performance like this is a pure exhibition, and although a physiologist may consider it a matter of interest to observe the changes which take place under such a system of starvation, it has no possible relation to nursing, and we cannot conceive that any nurses of recognised standing will be privy to such folly.

### SHORT ITEMS.

Extensive building operations are being carried out at the London Hospital, and the matron's office is temporarily moved to make room for the workmen. Among other improvements which will be welcomed by the nurses is a passenger lift. A spiral staircase is also being built, and the walls of the passages are being covered with white glazed tiles.

### Lectures to Nurses on Anatomy.

By W. Johnson-Smith, F.R.C.S., Principal Medical Officer, Seamens' Hospital, Greenwich.

### LECTURE I.—THE SKELETON.

Those who wish to gain an intelligent and really helpful knowledge of human anatomy should begin their instruction with a fixed idea that too much time and trouble cannot be spent over a study of the skeleton. "The student who knows his bones well, knows more than half his anatomy," was an oft-repeated saying of a well-known anatomist. It will very probably be found the most attractive, or perhaps the beginner might protest, the least unpleasant branch of the subject to which it is intended to devote this course of lectures. Osteology, as the study of the bones is called, has a wide range beyond its practical application to the work of the doctor and nurse, as it is essential to a clear comprehension of the natural history of both living and extinct animals, and, moreover, as is shown by the frequent presence of the skeleton in their works both of painting and sculpture, was evidently regarded by artists of old as a subject of æsthetic interest.

A casual view of a good plate or an actual specimen of the human skeleton is likely to cause some bewilderment as a result of the large number of the constituent bones, their exuberant variations of form, and their apparent intricacy in arrangement. The perplexity thus suggested may, however, be readily overcome by the considerations that the number of bones to be studied constitutes no real difficulty; that the variety of shapes may be reduced to four or five characteristic types, and that the arrangement of the bones is really very simple and not at all intricate.

It is impossible to give a precise answer to the question "How many bones are there in the human subject?" for the reason that their number varies according to the period of life. The child has more than its parents, as in the former there are several groups of distinct bones which, by the age of 20, are united together to form a single bone. There are in the fully-developed skeleton about 214 distinct bones, but to facilitate study the small and hidden bones of the ear, and some minute bones attached to the thumb and great toe may be excluded from present notice, and the total be given as 200. Of these 200 bones, 166 make up pairs and are arranged symmetrically on both sides, and the remaining 34 are single bones disposed for the most part in the middle line between the right and left halves of the body. Thus the number of bones to be studied is reduced to 117, and, moreover, in dealing with certain groups of bones as, for instance, the ribs and the bones of the toes and fingers, special attention need not be given to more than one or two specimens of each.

The teeth are not included in the skeleton as they are like bones only in their hardness, and differ altogether from them in their construction, their mode of growth, and with regard to the nature of the structures in which they are first formed.

The diversity of external form in the constituent parts of the skeleton may be simplified to some extent by arranging them in a small number of distinct groups. Many of the bones, especially those of the limbs, it will be seen, are elongated and columnar, others, like the shoulder-blade and the bones of the skull-cap, are expanded into broad and thin plates; others, again, like the separate bones of the spine, are of very irregular form; and, finally, there remain a few bones, many of these of small size, which usually present geometrical outlines, and are much shorter than most of the long bones. Thus, as is done in most text-books on anatomy, the bones may be classified as long, flat, irregular, and short.

The group of long, or as I would prefer to call them, tubular bones, is well represented by the bones of the limbs and the ribs; that of the flat or tabular bones by the shoulder-blades and the constituent bones of the top and sides of the skull; and that of the irregular bones by the vertebræ or bones forming the spine, and the bones of the face. Most of the so-called short bones will be found at the ankle and wrist.

In the study of the skeleton, it will be found convenient to begin with the backbone or spine, then to consider the

Fig. 1.

bones around the three large cavities of the body—the head or cranium, the chest or thorax, and the abdomen—and finally to describe the bones of the limbs.

In the study of practical anatomy it is very necessary to have a clear idea of what is meant by terms intended to express the relation of one part to another. There might, in the first place, be some confusion with beginners as to the precise meaning of such simple terms as external and internal, anterior and posterior. In studying the skeleton, and, indeed, the whole human organism, we are to assume that the body is placed in its normal erect attitude. Thus, even when the body is recumbent, the head is superior to the thorax, and the leg is inferior to the thigh. The terms external and internal are employed to express the relative positions of two parts to an imaginary plane—the so-called mesial plane—passing from the front to the back of the body, and dividing it into two lateral halves, which on their surfaces, and as regards the bones and some other structures, are perfectly symmetrical. Thus on each side the eye is internal to the ear. By the terms anterior and posterior are implied relative position with regard to the back and front of the body—the breast-bone being anterior to the spine. The terms *proximal* and *distal*, often met with in anatomical description, have opposite meanings. The former denotes that part of an organ that is nearer to, and the latter that at a greater distance from the trunk, the heart, or the centre of the body. Thus the shoulder is the proximal end, and the finger tips form the distal end of the upper limb, and the elbow is distal with regard to the shoulder, and proximal with regard to the wrist. These two terms are applied most frequently to bones, blood-vessels, and the intestinal canal. The terms *central* and *peripheral*, also having opposite meanings are used generally in the description of nerves, the former term implying at or in the direction of the brain or spinal cord, and the latter at or in the direction of the organ to which the ultimate divisions of the nerve are distributed.

### The Army and Indian Nursing Service.

The Committee appointed by the Secretary of State for War to consider the Reorganisation of the Army and Indian Nursing Service, consisting of the same members as the Committee on the Reorganisation of the Army Medical Services, has drawn up the following report:—

### SCHEME FOR THE REORGANISATION OF THE ARMY AND INDIAN NURSING SERVICE.

#### The Queen as President.

1. There shall be one Military Nursing Service for His Majesty's Army in the United Kingdom, India, and the Colonies, to be designated "Queen Alexandra's Imperial Military Nursing Service" (Q.A.I.M.N.S.). In this service shall be amalgamated the existing Army Nursing Service and the Indian Nursing Service.

2. Her Majesty Queen Alexandra shall be requested graciously to assume the Presidency of this Service.

3. The Nursing Service shall be under the immediate control of Her Majesty Queen Alexandra as President, and of a Nursing Board constituted as follows:—

President.—Her Majesty Queen Alexandra.

Chairman.—The Director-General, Army Medical Service, or an Officer nominated by him.

Two members of the Advisory Board, Army Medical Service, of whom one shall be a civilian.

The Matron-in-Chief, Queen Alexandra's Imperial Military Nursing Service.

Three matrons of large civil hospitals with medical schools.

One representative of the India Office to be appointed by the Secretary of State for India.

Two members to be nominated by Her Majesty the President, and holding office for three years.

4. Upon this Nursing Board the civilian members of the Advisory Board, Army Medical Service, and the matrons of civil hospitals shall be appointed by the Crown, on the advice of the Secretary of State, and shall hold office for a period of three years, renewable on expiration of the term of appointment

A matron of a civil hospital shall receive an honorarium of £26 5s. per annum while serving on the Board.

#### The Nursing Board.

5. The Nursing Board, of which three shall form a quorum, shall usually meet at fortnightly intervals. The minutes of the proceedings of the Nursing Board shall be laid by the Matron-in-Chief before the Advisory Board. It shall be in the power of the Advisory Board to refer back any point to the Nursing Board for reconsideration, and in case of a divergence of opinion between the Boards, the matter in question shall be referred to the Secretary of State.

6. Subject to the general control of the Advisory Board the Nursing Board shall be responsible for—

(1) Advising the Secretary of State upon the strength of the Nursing Staff of various grades requisite in each military hospital (including the hospitals for women and children attached to military stations), having regard to the character of the cases admitted, and subject to the proviso that as a general rule hospitals containing fewer than 100 beds will not be provided with a regular female nursing staff (*vide* paragraph 14).

(2) Defining the conditions under which nurses may enter the Service, the terms of their appointment, and the duties to be performed in the several grades of the Nursing Service.

(3) Dealing with all questions relating to the uniform and clothing allowance of the Nursing Service.

(4) Receiving and considering reports from Matron-in-Chief and the matrons of the various hospitals.

(5) Recommending to the Commander-in-Chief, for the approval of the Secretary of State, the appointment, retention, promotion, retirement, dismissal, and distribution of the members of the Nursing Service.

(6) Arranging for the selection and engagement of additional nurses, the organisation of the requisite nursing staff, and the appointment of Principal Matrons in case of war or epidemic.

(7) Advising the Secretary of State upon the formation of the Nursing Reserve of the Queen Alexandra's Imperial Military Nursing Service.

(8) Arranging for the periodical inspection of military hospitals as regards nursing efficiency.

(9) Submitting to the Secretary of State, through the Advisory Board, a scheme for the organisation and development in India of the Queen Alexandra's Imperial Military Nursing Service, which shall allow for adequate local control, subject to the general authority of the Nursing Board.

#### Duties of the Matron-in-Chief.

7. The Queen Alexandra's Imperial Military Nursing Service shall consist of—

(1) A Matron-in-Chief and Principal Matrons.

(2) Matrons.

(3) Sisters.

(4) Nurses.

8. All matrons, sisters, and nurses of the Queen Alexandra's Imperial Military Nursing Service shall be entitled to wear an appropriate badge, which, by special permission only of Her Majesty the President, may be retained by the wearer after leaving the Service.

9. The Matron-in-Chief shall have a seat on the Advisory Board, acting as a Member of the Board whenever matters concerning the Nursing Service are under discussion, and in her absence a Principal Matron shall take her duties.

10. The Matron-in-Chief shall be the medium of communication between the Director-General and the Queen

Alexandra's Imperial Military Nursing Service in all matters connected with that Service.

11. The Matron-in-Chief shall be responsible for keeping the Service records and confidential reports from the matrons of the various hospitals regarding the character, conduct, and efficiency of the sisters and nurses under their control.

12. The Matron-in-Chief shall keep herself acquainted with the administration of the Nursing Service in the various military hospitals.

13. She shall submit to the Nursing Board recommendations for the appointment, promotion, retirement, dismissal, and distribution of members of the service.

14. She shall be responsible for maintaining a sufficient staff of special nurses, detailing them for duty in cases of emergency, or for service in smaller hospitals.

15. She shall present every year to the Nursing Board a scheme for the annual leave of matrons and special nurses, and shall report to the Board the arrangements made by matrons for the annual leave of sisters and nurses.

Duties of a Matron.

16. Amongst the duties of a matron, to be defined in detail by the Nursing Board, shall be the following:—

(1) To recommend suitable candidates for admission to the service in accordance with the prescribed regulations.

(2) In conjunction with the Medical Officer in charge of the hospital to forward to the Matron-in-Chief such confidential reports with regard to the work and conduct of the nursing staff as may be required, and to make recommendations for retention, promotion, retirement, and dismissal.

(3) To be responsible for the general nursing arrangements of the hospital, for the due performance of their duties by the sisters and nurses, and for the maintenance of good conduct, efficiency, and discipline amongst all members of the female nursing staff. In conjunction with the Medical Officer in charge of the hospital to report upon these matters at stated intervals to the Nursing Board through the Matron-in-Chief.

(4) To exercise similar functions as regards the hospital for women and children in a station where such hospital exists.

(5) In urgent cases to provide, where practicable, for the nursing of women and children on the married establishment.

(6) To engage and dismiss the female servants appointed to attend upon the nursing staff, and to be responsible for their discipline, good conduct, and efficiency.

(7) To undertake the daily inspection of the nurses' quarters to ensure that they are clean, well ventilated, and kept in good order.

(8) To be responsible to the Medical Officer in charge of the hospital for sufficient supply, good condition, and cleanliness of the bedding and linen in the nurses' quarters and the wards under her nursing charge.

(9) To see that proper medical and nursing attendance is provided without delay for sick members of the nursing or female domestic staff.

(10) To arrange the annual leave of sisters, nurses, and female domestic staff, reporting thereon to the Matron-in-Chief.

17. A Principal Medical Officer shall report annually to the Nursing Board, through the General Officer Commanding, upon the conduct and efficiency of the matrons of hospitals within his district.

Duties of a Sister.

18. Amongst the duties of a sister in charge of a ward, to be defined in detail by the Nursing Board, shall be the following:—

(1) To be responsible for the cleanliness, ventilation, and good order of her ward and its annexes.

(2) To attend the Medical Officers in their visits to the ward, and carefully to carry out their orders with regard to the diet and treatment of patients.

(3) To see that the nurses and orderlies perform their duties punctually and efficiently, reporting any breach of discipline or neglect of duty on the part of a nurse to the Matron, and on the part of an orderly to the Medical Officer in charge of the ward, or in his absence to a warrant or non-commissioned officer of the Royal Army Medical Corps.

(4) To take part in the nursing of all patients seriously ill.

(5) To be responsible to the Matron and Medical Officer of the ward for sufficient supply, good condition, and cleanliness of the bedding and linen, and for the personal cleanliness of the patients.

Conditions of Admission to the Service.

19. Amongst the conditions under which nurses may enter the Service, and the terms of their appointment (to be defined in detail by the Nursing Board), are the following:—

(1) A candidate must be of British parentage, be between 25 and 35 years of age, and possess a certificate of not less than three years' training and service in medical and surgical nursing in a civil hospital recognised by the Advisory Board. She shall be required to satisfy the Nursing Board that as regards education, character, and social *status* she is a fit person to be admitted to the Queen Alexandra's Imperial Military Nursing Service.

(2) If provisionally accepted, she shall be placed on probation for a period of three months, at the end of which time, if her work and conduct are reported to be satisfactory by the matron of the hospital, she may, after having been medically examined, enter into an agreement binding herself to three years' service in the Queen Alexandra's Imperial Military Nursing Service, and undertaking to conform to the rules and regulations of the Service. The agreement shall be dated from the time at which the nurse was provisionally accepted, and may, on the recommendation of the Commander-in-Chief, be terminated at any time by three months' notice from the Secretary of State, or in case of grave breach of discipline or misconduct, without notice.

(3) On the expiration of her three years' term of service a nurse may be permitted—

(*a*) To retire from the service.

(*b*) To continue in the Service as a staff nurse, with an agreement terminable at any time by one month's notice on either side.

(*c*) To join the staff of special nurses under the orders of the Matron-in-Chief, with an agreement terminable at any time by one month's notice on either side.

(*d*) To offer herself for promotion to the post of sister, undertaking to serve for at least one year, an afterwards under an agreement terminable at any time by one month's notice on either side.

(*e*) To enter into a fresh agreement for service, as nurse or sister in India or elsewhere abroad, for a period of three or five years, according to climate.

20. All present members of the Army and Indian Nursing Service, and members of the Army Nursing Reserve who have been in military employment during the war in South Africa, shall be eligible for appointment in the Queen Alexandra's Imperial Military Nursing Service if recommended by the Nursing Board. Should any question arise as to their *status* in the Queen Alexandra's Imperial Military Nursing Service the Nursing Board shall report thereon to the Advisory Board, and the recommendation of the Advisory Board shall be submitted to the Commander-in-Chief, whose decision shall be final.

21. Any present member of the existing Services, who is not retained in the Queen Alexandra's Imperial Military Nursing Service, may be recommended for a gratuity of one month's pay for each year of service, if she is not entitled to a pension; and any member who may decline to accept the new terms of employmen t shall be allowed to serve upon the terms of her present engagement.

THE QUESTION OF PAY AND ALLOWANCES.

22. PAY.

(*a*) Nursing Staff—

Matron-in-Chief, £250 a year, rising by annual increments of £10 to £300, and lodging allowance.

Principal Matron in India, £230 a year, rising by annual increments of £10 to £280, and lodging allowance.

Principal matron, £110 a year, rising by annual increments of £5 to £160.

Matrons, according to size of hospital, £70 to £100 a year, rising by annual increments of £5 to £120 to £150.

Sisters, £37 10s. a year, rising by annual increments of £2 10s. to £50.

Nurses, £25 a year, rising by annual increments of £2 10s. to £35.

(*b*) Female servants—

£15 a year, rising by annual increments of £1 to £20.

23. ALLOWANCES.

(*a*) Nursing Staff—

Home station, board and washing, 15s. a week.
Station abroad, board and washing, 21s. a week.
Station abroad, washing, 3s. 6d. a week.
Home station, uniform, £6 per annum.
Station abroad, uniform, £7 per annum.
Home and abroad, clerks, £2 per annum.

(*b*) Female servants—

Board and washing, 10s. 6d. a week.

24. Allowances at the recognised scale shall be given for Indian and Colonial service.

25. The regular annual leave of members of the Queen Alexandra's Imperial Military Nursing Service in Home Stations shall be as follows:—

Matrons, 6 weeks.

Sisters, 5 weeks.

Nurses, 4 weeks.

Leave at stations abroad shall be granted on the military system.

26. It is desirable that all members of the Queen Alexandra's Imperial Military Nursing Service should be eligible to apply for a pension at the age of 50 years, and should be retired at the age of 55 years. Rates of pension shall be according to the scale laid down in Article 1233, Royal Warrant for Pay and Promotion.

### To Nurses.

We invite contributions from any of our readers, and shall be glad to pay for "Notes on News from the Nursing World," or for articles describing nursing experiences, or dealing with any nursing question from an original point of view. The minimum payment for contributions is 5s., but we welcome interesting contributions of a column, or a page, in length. It may be added that notices of entertainments, presentations, and deaths are not paid for, but, of course, we are always glad to receive them. All rejected manuscripts are returned in due course, and all payments for manuscripts used are made as early as possible after the beginning of each quarter.

### The Nurse's Never.

BY A MEDICAL LECTURER ON NURSING.

Never imagine that you are responsible for the treatment of your patient. What you are responsible for is the effective carrying out of the treatment. As Epictetus says of life's drama, "You may choose to play your part well or badly, but you do not choose your part. That choice is made for you by the author of the play."

Never plead that you "did it for the best." Your only plea is that you did as you were told.

Never let "feeling run away with reason in her arms." It is useless to say "I felt I ought to do something for the patient," when you knew that you should have stood by and waited.

Never wait to be spoon-fed with knowledge. If you have not passed the stage of being nursed intellectually you are not qualified for your post.

Never be ashamed to say "I don't know." If it is something knowable you will be led to learn it; if it is unknowable you may lay to heart the saying that "Many things in this life must be left abrupt."

Never omit the opportunity of learning from your own mistakes. "The man who never made a mistake never made anything."

Never confuse saying with doing. Some men never say a foolish thing and never do a wise one.

Never express an opinion unless you are asked to do so.

Never be unpunctual. And remember it is nearly as bad to be too early as it is to be too late.

Never be slovenly in anything, even in handwriting. A nurse has no more business to write illegibly than she has to appear in *déshabille*.

Never argue. "Controversy equalises wise men and fools, and the fools know it."

Never neglect your own mind and body. "She went clean again the Scriptur', for that says, 'Love your neighbour as yourself;' but I said, 'If you love your neighbour no better nor you do yourself, Dinah, it's little enough you'd do for him.' You'd be thinking he might do well enough on a half-empty stomach." (George Eliot in "Adam Bede.")

Never be over-familiar either with persons or diseases Familiarity breeds carelessness as it breeds contempt.

Never gossip. Much gossip is made up consciously or unconsciously of half truths; and "a lie which is half a truth is ever the blackest of lies."

Never grumble. If you must say "plain words" remember you can say the plainest words with the finest manner If you are debarred from speaking, then work it off; 'there's nothing but what's bearable as long as a man can work."

Never appear despondent, especially if the case you are attending is hopeless. A patient ought never to read his death-warrant in your face.

Never rest content with low aims. "If you aim at the moon you will never land in a blackberry bush."

Never stand if you can sit, and never sit if you can lie down. This is a woman's golden rule for long life and good health.

Never lose your self-control. The best way to acquire self-control in life is to "always do the thing you are afraid to do."

Never make comparisons between cases unless you know all the facts. Do not imagine your patient must die, because another of a different sex, of a different age, in a different place, and suffering from a different disease, died.

(*To be continued.*)

### The Congress of Nurses at Buffalo.

By Our Own Correspondent.

Buffalo, N.Y., September 16th.—Probably the largest gathering of nurses ever held in the world opened a week's session in the Woman's Union Building in this city to-day. It is the third congress of nurses, but the first congress holding an independent session, the two former having been held as sub-sections of other world-gatherings of women. The first was in connection with the Congress of Medicine in Chicago during the World's Fair in 1893, and the second as a part of the International Council of Women held in England two years ago. But this week's congress is purely and solely for and by nurses, of which 102 separate societies are represented, with a total membership estimated at 15,000. Of the societies composing the congress, there are 20 from England and her colonies, one international, two national United States, and 99 guilds, alumnæ, and hospital associations from the United States. The following are delegates and the organisations they represent: — Mrs. Bedford Fenwick, President of the International Council of Nurses; Miss Annie Arkle, delegate from the Indian Nursing Service, India Office, Whitehall, S.W.; Miss Sophia Cartwright, delegate from the Registered Nurses' Society, London, England; Miss Susan McGahey, delegate from the Australasian Nurses' Federation, Australasia; Miss M. Mollett, of the Matrons' Council of England; Miss Louisa Stevenson, of the National Union of Women Workers of Great Britain; Mrs. Rebecca Strong, of the Royal Infirmary, Glasgow, Scotland; Miss Emilie M. Waind, of St. Bartholomew's League, London, England; Miss C. J. Wood and Miss Amy Hughes, representing the Queen Victoria Jubilee Institute for Nurses, Colonial Nursing Association, Workhouse Infirmary Nursing Association, School Nurses' Society, Guy's Hospital Training School for Nurses, Guy's Trained Nurses' Institute, Leicester Infirmary, Maternity and District Nurses' Home (Plaistow), Asylum Workers' Association, Midwives' Institute and Trained Nurses' Club, Incorporated Society of Trained Masseuses, and the Nurses' Hostel.

### The International Council of Nurses.

To-day (Monday) the International Council of Nurses was called together by the president, Mrs. Bedford Fenwick.

Mrs. Fenwick, who wore a royal blue tailor costume, with an immense white feather boa and large white hat, in addressing the Council said: The work which lies before us in the perfect organisation of an International Council of Nurses may well impress us with its magnitude. We have written down its constitution, a constitution pregnant with, and powerful for, good, but we have to make that constitution live. To do this we must inspire it with the vital force of a fine purposeful spirit. Hence, work must be our watchword. We have inspiration and effort but we also need order. In the formation of the International Council of Nurses its founders have looked well to its organisation. The vote covers all. They have therefore chosen graduate suffrage as the foundation on which to erect their stately pillar of international professional co-operation, and have thus based the constitution on the fundamental principle that a free and therefore a progressive community must be self-governing. The organisation of the International Council is as simple as it is sure. The graduate nurse combines to form alumnæ associations; by delegation, these societies co-operate to form a national association. The National Associated Alumnæ, in conjunction with the Superintendents' Society, federate to make a National Council, and the National Councils are eligible for affiliation with the International Council of Nurses. Thus, through gradual delegation, we provide that every graduate nurse becomes articulate in this International Council of Nurses. We have, in short, secured to our members professional suffrage, and order will thus be evolved out of chaos. And yet, in making our council mechanically perfect, let us remember that the vital force is of the spirit and not of the letter of the law. In a society which embraces members of different race and creed, we must, while maintaining inviolate certain broad general principles which form our common bond of union, permit, nay foster, individuality in detail—authorising each country to apply these principles in a manner best suited to its own needs. In like manner every national council will do well to encourage and develop the individuality of its members, for only so shall we utilise to the full the correlation of our forces, and make our council a progressive power for good. Diversity of opinion is the very salt of life, and we shall do well to encourage rather than depreciate its expression. The task to which we must first devote all our energies is the building up of national councils of nurses in every land. Into these councils should be gathered, through various associations for mutual help and professional progress, every individual graduate nurse; and the chief work in European countries for many years to come will be the education of these graduates in the immensity of human responsibility, which includes their duty towards their neighbour other than their patient, and their duty to the State.

### The World's Nursing.

Reports of the status of nursing in various countries were an interesting feature of this session. Brief abstracts are given:

Mrs. Fenwick, for Great Britain and Ireland, reported that the general course of training is three years, in some hospitals in Ireland two, and in both countries the system of paying probationers is enforced to some extent from three months to a year; and that organisation of nurses is quite general.

Miss McGahey, for Australia, said that there were several good hospitals there with a well-defined three-year course as standard, and systematic schedules of theoretical work. There are two nurses' organisations in form to enter the International Council.

Mrs. O'Neill, for New Zealand, reported three-year courses in the hospitals with an eight-hour system, schedules of theoretical teaching, no graduate societies.

For South Africa, Miss Margaret Breay told of a hospital in Uganda, on the Victoria Nyanza, which had a three-years' course; one on the Island of Likoma, where the nurses were all certificated British nurses; and a similar one in Zomba. There were several on the northern seaboard, she said, and one at Cairo in which natives, men and women, are trained. On the east coast there are English, French, German, and native hospitals. The English train the natives, teaching no theory, but giving good practical training. Of all countries, South Africa alone has State registration, the effect of which, on the whole, is good. There is no organisation outside Cape Colony.

Miss Milne reported for Tasmania three-year courses, with theoretical and practical courses. No organisation, but nurses preparing to join Australasian Nurses' Association.

Nursing in France is in a deplorable state, according to Dr. Anna Hamilton. It is done by religious orders and much behind the times scientifically. One hospital, the Protestant House of Health for Bordeaux, since May, 1901, has been in charge of Dr. Hamilton, who is placing trained nurses in position to teach, and will establish a training school. No organisation in France.

Miss C. A. Bastide Bairslag reported many excellent hospitals in Holland. The three-year course is general, with a few exceptions, and theoretical teaching is given. There are several associations of nurses, two of them publishing journals.

Miss Amy Turton, for Italy, said that up to 1892 there were no training schools. Nursing had previously been done by religious orders. Now there are schools of nursing in Rome, Naples, and Florence. The period of training is two years, with theoretical and practical work.

From Froken Gina Krog came Sweden's report. The period of training is one and a half year, with an agreement on the part of the nurse to give another year after leaving, making practically a three-years' course. No independent organisation of nurses, but the schools and hospitals form societies to look after the nurses' outside work and welfare. The nurses have a benevolent society called the Frederika Bremer.

Denmark, reported by Mrs. Gordon Norrie, has a large number of well-appointed hospitals, the term of training varying from six months to 15 months. After this the nurse remains on salary as assistant nurse, with hospital training continuing, for periods that vary. The nurses have one organisation, the Danish Nurses' Council.

For Greece, Mrs. Fenwick said that nursing as a profession is just beginning to receive a stimulus fostered by the Queen. So far English nurses have done all work of importance in the Greek hospitals. Training is being established, but so far only the foundation has been laid.

Brazil, Miss J. A. Jackson said, is 100 years behind the times. There are no training schools, and nursing is done by religious orders or graduate nurses from England.

In Cuba, Mrs. Quintard reported the establishment of training schools for women by American nurses. The schools now begun have a two years' course, to be extended later. There are no graduates yet, and no organisation of course.

Canada and the United States were reported respectively by Miss Snively, of the Toronto General Hospital, and Miss Dock, of New York. Conditions were similar, the three years' course gradually taking the place in all hospitals of the two years'. Practical and theoretical work given and the graduates fairly well organised.

### The Presidential Address.

When the Congress opened formally on Wednesday morning the assembly hall was crowded. Miss Isabel McIsaac, the Congress president, sat in the centre of the stage, and around her were the other officers of the league and the speakers of the day.

The Hon. Conrad Diehl, Mayor of Buffalo, who was to have welcomed the Congress for the city, was absent, having attended the funeral of the murdered President in Washington; and Mr. Constantine, his secretary, in his stead, delivered an address of welcome.

Mrs. George W. Townsend, president of the Woman's Union, in whose building the meetings were held, also delivered an address of welcome in behalf of the women, and then followed the address of Miss McIsaac, She said:—

It is with extreme pleasure and appreciation I respond in behalf of the Congress to the cordial Buffalo welcome of Mrs. Townsend and his Honour the Mayor, who have honoured us not only by the warmth of their reception, but by their recognition of our profession. I have the additional pleasure in extending the welcome of American nurses to the foreign delegates and representatives whose presence and participation in this Congress will contribute in so marked a degree to its interest and success. In approaching the discharge of my duties as presiding officer of this third Congress of Nurses, I beg to express my appreciation of the generosity by which I have been called to such an honour. If the phenomenal growth of nursing is any indication of its righteousness, then who can doubt our future? Small wonder that our pioneers, some of whom are still with us, express themselves as sometimes awed by the mighty impetus of the ball they started rolling scarcely more than a generation ago. To our English colleagues we of the United States owe more than we can ever repay, and if in our swift American fashion we have broken from their leading strings and made paths for ourselves we none the less acknowledge our indebtedness with gratitude. Our first international gathering in Chicago in 1893 was marked very distinctly by the making of acquaintance, which sounds rather insignificant, but on second thought assumes its proper place, and we realise that it signified a tremendous force in nursing affairs. The exchange of experiences suddenly roused many women to the fact that the deficiencies and difficulties of their work were peculiar to the whole nursing profession and not to one school or hospital, and from that meeting originated nearly all the progress which has gone on since, in America at least, and we will devoutly hope that from this Congress as much that is good and great may come. Any number of the problems up for discussion, then, still confront us in both continents—the uniform requirements for admission to our schools, the uniform curriculum, what shall constitute a trained nurse, state registration, local and national organisation, a code of ethics, and many minor questions. In America the training course lasting from two to three years is nearer an accomplished fact than any other question, and, while the curriculum is far nearer uniformity than eight years ago, there is still much to be desired. The question as to what constitutes a trained nurse seems further from settlement in this country than at any time before. In our nervous energy and haste to embrace all things new, and to get to the end by a short cut, we often sacrifice both quality and thoroughness to speed; and in no other work is this more glaring than in the enormous increase of so-called training schools, which have neither educational nor moral right to exist. We will listen with much interest and eagerness to our foreign delegates upon this subject, for it is one of tremendous gravity to our profession. The establishment of a chair of Hospital Economics in Columbia University has been one of our most important undertakings, originating with the nurse, Isabel Hampton Robb, who has done more for our profession in America than any other one woman. The Columbia course will undoubtedly be a most valuable leaven for the whole lump, and I may say with no fear of giving offence that the superintendents themselves know better than anyone else the great need of better teachers. The organisations for nurses all over the world have developed wonderfully, and, while we occasionally hear expressions of discouragement, we should not forget that we have learned much by contact, and see our deficiencies now far more than formerly. A topic new to the nurses of the United States since our first meeting, although an old one in England, is army nursing, a huge problem undertaken here in an emergency and one in which we sadly acknowledge we have not always done ourselves credit, nor, perhaps, always given credit where it may have been due. An undertaking of which we are justly proud is the *American Journal of Nursing*. To paraphrase our great Lincoln, 'a journal of nursing, for nurses and by nurses,' the work of which has been entirely done, until within a few weeks, by nurses hard-worked in other lines, which is a monument to the courage and devotion of American nurses. Again, I beg to express our thanks to our cordial hosts of Buffalo, and to extend to the distinguished guests within our gates who share with us this undertaking the hand of fellowship and felicitate them upon their achievements in our great profession.

### HOSPITAL ADMINISTRATION IN GREAT BRITAIN.

This was the first subject on the programme, and papers were read by Miss Isla Stewart, superintendent of nursing in St. Bartholomew's Hospital, London, and president of the Matrons' Council of England, and by Miss Wilhelmina J. Mollett, superintendent of nursing, Royal South Hants and Southampton Hospital. Both papers were technical, but gave the American nurses a very clear idea of the practical working order of English hospitals.

Miss Stewart, in the course of her paper, said: The broad lines of administration are much the same in a large majority of the hospitals of Great Britain and Ireland, which are either endowed or partially, or wholly, supported by voluntary contributions. Many hospitals have as their highest representative a patron or president, who in quite a large number of cases are Royal personages, and they are by no means ornamental, as their patronage implies not only a personal contribution to the funds, but very material assistance in attracting the gifts of the public, who feel that a certain guarantee of efficiency and probity is given by the use of the name being allowed. Nor is this a misplaced belief, as every care is taken in the way of inquiries and inspection to prevent the name of any of the immediate Royal family being used in connection with any institution the general management of which is open to question. The subscribers elect the governors from among themselves. These form a court, which meets annually, half-yearly, or quarterly. They appoint a sub-committee, frequently known as the House Committee, which meets monthly or fortnightly, and in nearly all the large hospitals there is also a weekly board, empowered to deal with minor matters. The *ex-officio* chairman of every board and committee is the treasurer, elected by the governors, in some hospitals for life and in others annually. There are also three or more trustees, who are members, *ex-officio*, of the committees. In a few hospitals there are women on the governing board. The Royal Infirmary in Edinburgh is the most important hospital where this is the case. Two women there have been elected to serve on the committees. The Royal Infirmary in Glasgow has followed its example, but they have placed not only two women on their committee, but two working men. In many cases, indeed, in almost all provincial hospitals and in Scotland and Ireland, the medical staff are represented on the board by two or more members. When this is not the case the medical staff form themselves into an advisory committee of their whole number, and are consulted by the lay governing body on all matters which affect the interest or the well-being of the patients.

In many of the London, Edinburgh, and large provincial hospitals, the chief resident authority is a superintendent, who may belong to the medical or legal professions or may be an army man or a civilian of tried business capacity. He may be styled the clerk, house governor, or superintendent. His duties are numberless, and comprise the decision of questions, chiefly administrative, which may involve considerable interest or be very unimportant. Under him are the heads of departments—the matron, head of the nursing and domestic department; the steward, head of the department which includes payment of wages, bills, catering, recording admission and discharge of patients and the control of attendants and porters; the clerk, who has charge of the actual structure of the building and control of the carpenters and plumbers. In general each official reports individually, in writing, to the weekly and fortnightly board, but this is not always the case.

There is a very large number of important infirmaries, originally under the Poor Law Board, the powers of which were transferred to the Local Government Board by Act of Parliament in 1871. These are entirely supported by rates. Each parish, or group of parishes, supports its own institution. In England and Ireland guardians, and in Scotland county councillors, who may be either men or women, are elected by the ratepayers and hold office for three years. They attend a fortnightly board, which deals with all the matters which would be brought before the governors of the voluntary hospitals. Supreme authority is vested in the Local Government Board, and all matters of any importance must be satisfied by it. The medical superintendent is in all cases the highest resident authority. The matron and steward act under his authority, and, although he may allow them a fairly free hand, he can call them to account when he considers it necessary. The matron is nominally the head of the nursing staff, but, as each nurse can appeal on any matter to the medical superintendent, her authority depends largely upon him. These hospitals are periodically inspected by inspectors employed by the Local Government Board, and who report to the Board directly. The hospitals for infectious diseases are also under the Poor Law, and are supported by the rates. In London they are governed by the Metropolitan Asylums Board, which is composed of representatives from the various boards of guardians, but one-third of the whole number of members are nominated by the Local Government Board. The system works well, and with marvellously little friction. The chief fault lies in a certain lack of minute discipline, which is not so apparent now as in the past, and which may have been largely due to the fact that the matrons were untrained or partially-trained women, often with little or no education, and who were given only partial control over the nursing staff—that is, when they were off duty. Now that both the guardians and the Metropolitan Asylums Board are appointing women of education who are fully-trained nurses, the friction between the medical superintendent and the matron is disappearing, though even now the discipline is not quite so perfect as in the general hospitals. The control over the nurse is nothing like so absolute. She signs no contract on entering, and has no training certificate to look forward to. The medical staff in the large hospitals in London, Edinburgh, Dublin, and in the important provincial towns, consists of a consulting, a visiting, and a resident staff. The consulting physicians and surgeons are mainly those who, having reached a prescribed limit of age, have retired from the visiting staff, their duties being merely nominal; the senior visiting staff and the physicians and surgeons, who pay periodical visits to the hospitals and have a certain number of beds allotted to their care. They visit on certain days at regular hours in London, usually three to four days a week at 1.30 o'clock. The junior visiting staff see the out-patients and have one or two days allotted to each of them. There is a still more junior staff who see the casualties every morning; and there are the heads of the various departments—gynæcological, ophthalmic, aural, throat, dental, orthopædic, and electric. There are two or more registrars, who superintend the recording of cases by the students, and a senior and junior anæsthetist. The resident medical staff consists of a house physician and surgeon to each of the visiting staff and to the heads of the gynæcological and ophthalmic departments. In some hospitals, notably St. Thomas's, there is a principal medical and a principal surgical officer, who hold their appointments for three years; but in the majority of hospitals the house staff is responsible for the patients during the absence of the visiting staff. Perhaps the most remarkable change in the administration of hospitals of late years has been the enormous increase in the number of nurses and in the expense of the nursing department, which in some hospitals seems to have reached an excessive point, and indicates the necessity for some competent authority to define the requirements of hospitals in this matter, having regard to the size of the institution and the chronic or acute nature of the cases received. The staff of the various large hospitals vary in proportion to the number of patients to an almost incredible amount. In the London Hospital, where there are 776 beds, with an average of 659 occupied, the whole staff, including the matron and her assistants, is 313, which is one nurse to about two and one-third patients on the whole number of beds, and just over one nurse to two patients on the number of beds occupied. In King's College Hospital, where there are 221 beds, and an average of 168 occupied, there is a nursing staff of 69, which brings the proportion of nurses to patients to very much the same as the London Hospital. In St. Thomas's Hospital the beds number 570, the inclusive staff 161, which makes the proportion quite one to three and one-quarter patients. In the Edinburgh Infirmary, where there are 780 beds, with an average of 711 occupied, the nursing staff is 195, making an average of one nurse to four beds, and rather less per patient. The Royal Infirmary of Glasgow gives almost the same proportion with 582 beds, an average of 555 occupied, and a nursing staff of 142. The Western Infirmary, Glasgow, with 420 beds, and a nursing staff of 128, gives a little better proportion. In the infirmaries and hospitals under the Poor Law, the proportion of numbers is curiously different. In Bethnal Green Infirmary, the number of beds being 669, with an average of 520 occupied, the number of the nursing staff is 80, giving one nurse to six and one-quarter patients. In the Lewisham Infirmary, the number of beds being 400, with an average of 250 occupied, the average is one nurse to almost seven patients. In the Poor Law Infirmary, Birmingham, with 1,540 beds, and an average of 1,131 occupied, the nurses' staff gives an average of one nurse to ten patients. The difference in proportion of nurses and patients in hospital and poor law infirmaries does not imply a corresponding lack of efficiency. A larger number of patients per nurse may be efficiently attended to in a poor law infirmary than in a hospital, in consequence of the chronic character of many of the patients. In the large hospitals the average cost of each nurse ranges from £40 to £63 per annum; in the smaller hospitals it ranges from £33 to £58. In London hospitals the majority have an expenditure of over £70 per bed. The Scottish hospitals are about £50 a bed, the Irish £40, and the provincial about £50. In London there are about six general hospitals that have an expenditure of over £100 a bed. The administration of the funds of the large hospitals is becoming more and more difficult as the expenses of each department increase. There must be some limit to the money obtainable for charity, and there should be some limit more stringent and effective on those who seek for free medical aid. There have sprung up in late years admirable societies for collecting and distributing money for the use of hospitals. The governing boards of hospitals are now largely composed of business men who have experience in the handling of great sums of money, and who give their time most ungrudgingly to the service of the hospitals.

### COUNTY HOSPITALS.

Miss Mollett's paper dealt with the county hospitals, and was as follows:—Ladies,—I rise with some diffidence to speak after Miss Stewart's able paper, dealing as it does with all the chief points of interest in hospital administration, and my only excuse is that as a matron of a very different, though very important, class of hospital, that is to say, large county hospital unconnected with medical schools, it may not perhaps be out of place if I commence with a few remarks regarding their importance from a statistical point of view in England. I am excluding the hospitals of Scotland, Ireland, and Wales, and all special hospitals. London contains 12 general hospitals with medical schools attached, containing an aggregate of 4,674 beds. The provinces have 12 medical schools with an aggregate of 3,075 beds. Thus the total number of beds in general hospitals in England where regular clinical instruction is given is 7,749. But there are further a very large number of general hospitals, varying much in size, which have no medical schools attached to them, whose total number of beds, 14,974, is nearly double the amount of those devoted to clinical instruction. Fifty of these contain 100 beds and over, and have a total of 7,526 beds, or nearly as many as the London provisional medical schools combined. Sixty-four have from 50 to 100 beds with a total of 3,472, while there are no less than 203 hospitals containing less than 50 beds, of which 60 have less than 10 beds, with a total of 3,976. The figures are taken from the Medical Directory. All the above are voluntary, hospitals supported by subscriptions or endowments. None of them are aided by Government or are rate supported. Very few indeed of them receive a small proportion of paying patients—in fact, they practically do not receive them. Except in certain primary matters it is not possible, in my opinion, to compare the management of a county hospital in detail with that of a hospital having a medical school attached. The essential virtues of order, discipline, obedience, and the subordination of the female staff in disciplinary matters to the female head are the same in both, but in detail they differ. The highest authority in a county hospital supported by voluntary contributions is always the governors, the donors, or subscribers in council assembled. The amount given or subscribed to become a governor varies, but the outcome is the same, the formation of a large body with voting powers meeting about once or twice a year to appoint committees and vote extraordinary sums or changes in the by-laws of the institution. They elect the management, financial and executive committees for the year, the chairman, etc., and these practically carry on the business of the hospital, appointing the executive officers, and being generally responsible to the governors for the efficiency and economy of the place. It is here that both the strength and weakness of hospital government lies. While on the one hand the management of the hospital is kept in touch with public opinion, on the other hand the proper government of the hospital is apt to be disturbed by well-meaning gentlemen who, having no knowledge of the real needs and requirements of hospital wards, lay themselves open to the influence of popular or strong executive officers. The honorary medical staff have representatives on the various committees, the manner of this representation varying slightly in different hospitals. The executive administration in my hospital, which contains 130 beds, and which I am following in this sketch, falls into three departments, and I hold that it is the proper balance of power between those three departments. They are the secretarial, the medical, and the nursing and domestic. The secretary has charge, under the finance committee, of the financial affairs of the hospital, the collecting of subscriptions, banking business, balance-sheets, etc. He is a non-resident officer. The principal resident medical officer is the senior house surgeon, who, working under the honorary medical staff, acts for them in their absence and is in medical and surgical charge of the patients; but as he is besides the resident medical officer for the committee, he holds—and rightly—a very important post with regard to the patients. He is, generally speaking, responsible for the admission and discharge of the patients, and for their treatment in the absence of the honorary staff, but he is not an administrative officer as regards the nursing and domestic staff. One of the greatest difficulties in a county hospital is in securing a suitable man for the post of house surgeon. It is essential that house surgeons should be thoroughly good professional men and men of common sense, who work their way into their post without offending against all its conservative instincts. For they come of course from various medical schools. All think their own school perfection and are often a trifle scornful of their predecessor's methods. But the main point is that they should not be slack but keen men for their work, and perhaps from a matron's point of view, that they should not be too susceptible to the charms of attractive sisters and probationers. The matron's department includes the nursing and domestic staff, the food, the laundry, and the general management. She either selects the sisters or recommends candidates to the board, engages probationers and servants and superintends their work, for which she is responsible. She gives the orders to the assistant matron and to the cook and supervises more or less directly the food for patients. Her position is a combination of superintendent of nursing with that of matron. I will add that it is essential, if she is to carry it out efficiently, that she should herself, as in my case, be directly responsible for it to the committee and not to any other official. Good nursing depends so much on good domestic management and is so intimately connected with it that the two should certainly be under the same control. The nursing staff is divided into sisters at the heads of wards, a night superintendent and probationers who train for three years and are stationed on night or day duty. Their average daily time on duty is about ten hours. I see no reason in a county hospital, why that time should be lessened. Wardmaids are attached to the wards to do the roughest of the work, but there still remains a fair, but not an undue amount of ward cleaning to be done by the probationers. The probationers receive lectures from the visiting medical staff and classes from the matron, and have to pass examinations before receiving their certificate of training. The whole scheme of county hospitals resolves itself into a body of subscribers, appointing committees for a given period, who in their turn control and regulate the hospital in accordance with certain rules and by-laws, and the rule of most of these hospitals is that they are intended only for "fit objects of charity." This rule is carefully guarded by the medical profession, among whom the idea of pay wards attached to a general hospital, which seem so usual here, is very generally regarded with great suspicion and disfavour. It must, however, be owned that they solve a most serious difficulty with regard to the poorer middle classes, who are of all people the worst off in England in illness. Above everything else these hospitals are pre-eminently intended for the sick poor, for their comfort, their convenience, their medical and surgical treatment—and the first and finest lesson they have to teach to the nurses trained in their work, is that nothing, not the nurses' instruction nor convenience nor comfort, is so important as the welfare of the patients confided to their care.

Miss Maud Banfield read a paper on "Hospital Administration in America," in which she pointed out the defects of the business methods of some American hospitals, and criticised the boards of managers and men superintendents for incompetency. The discussion which followed showed that while these conditions occasionally exist in isolated sections of the country, especially in hospitals where politics are influential in the management, they are the exception and not the rule in America.

"Hospital Administration in Relation to Training Schools" was the subject of a paper by Miss Riddle, assistant superintendent of nursing in Boston City Hospital.

"Women on Hospital Boards" formed the subject of an address by Mrs. Hunter Robb, of Cleveland, vice-president of the Congress and late superintendent of nursing in Johns Hopkins, and Illinois Training School for Nurses. This precipitated a lively discussion both for and against women as members of managing boards, but with the majority plainly against.

Miss Louisa Stevenson, of Edinburgh, made the hit of the afternoon in her view of the matter. Miss Stevenson is a delegate from the National Union of Women Workers of Great Britain. She was especially invited to speak on this subject. Miss Stevenson is a quaint, dignified figure, and her wit delighted the American women.

"For many years," she said, "I was of the opinion that much of the work in hospitals would be left undone if there were no women to attend to it. For five years, the limit of a term, I served on the board of the Royal Infirmary of Edinburgh. The first year I was alone—one woman among nineteen men—but the second year another woman was appointed. Throughout the whole term I was on very friendly terms with both the nurses and matron, and the men members of the board, so that I know women may be members of hospital managing boards and get along absolutely without friction. Miss Stevenson said that she was a believer in men and women working together as they were intended, and had therefere less faith in the advisability of boards composed entirely of women. "I do not know how it is in America," she continued, "but in Great Britain it has been my experience that there is not such a superabundant amount of executive ability among men as to make it advisable to do away with any that women may have. They must work together to get the best results, and in that view of it I want to emphasise that there is extreme benefit in women managers. After five years' experience, I still assure you that there is work in the hospital boards that will be left undone unless women take it up."

At the afternoon session Miss Kimber was announced to speak on the subject of "Nurses' Co-operative Societies," but she was absent, and Miss Sophia Cartwright, delegate from the Registered Nurses' Society, read a paper, in which she gave a concise account of the distinctive features of that society.

Miss Emilie Waind, delegate from St. Bartholomew's League, next described that organisation.

A discussion followed concerning the value of nurses societies, and Miss Isla Stewart, founder of St. Bartholomew's League, was called upon. Miss Stewart said that the league was purely for pleasure, and the members did not do much else at the meetings except enjoy themselves. "We are believers," she said, "in being, when off duty, away from it. We have one business meeting in a year, and all the others are social, at which we drink as much tea and coffee and eat as much cake as possible. Our league is very frivolous compared to your more serious working societies."

At this point Miss McIsaac left the chair. "I can't resist that," she cried. "Our English friends will go away thinking we care only for work. We care for fun too. Our Alumn Association in Chicago is frivolous too. And I want to speak of one feature of it that we especially enjoy, and which others may like to follow. It is on our graduating day we all come together and have an alumn banquet, with the graduating class as guests of honour."

Miss Snively, of Toronto, and Miss Hay, of Illinois, also entered the discussion, and the session concluded with a paper by Miss S. B. McGahey, on "Nurses' Federation of Australasia" which we are compelled to hold over.

### The Care of Epileptics.

### HINTS FOR PRIVATE NURSES BY A TRAINED NURSE.

It has occurred to me that a few hints based on long experience in the care of epileptics at home might prove useful to private nurses. There are many points in connection with the nursing and daily attendance on these distressing cases which cannot be learned in the hospital ward, where only the most acute forms of epilepsy are met with, and these but rarely. It is not proposed to deal with the "first aid" aspect of the epileptic, nor to describe the actual nursing of an acute case confined to bed, but to give some useful hints to the private nurse as to the treatment of a chronic case, and to lay down some guiding rules with regard to the hygiene and personal care which has proved beneficial in epilepsy. A private nurse is often sent to a patient suffering from the disease without any previous experience in the care of epileptics, beyond a very few acute cases she has met with during her hospital training. The nursing text-books tell little or nothing of a nurse's duties in such cases. Each nurse, therefore, has more or less to evolve a system out of her own understanding, since the chronic epileptic usually has no strict code laid down by his doctor for the nurse to follow. These cases are governed by general principles only. Having seen so many nurses groping, as it were, in the dark when entrusted with an epileptic, I venture to offer these points out of my own experiences.

### Epileptics not Easy to Manage.

The happiness and well-being of a confirmed epileptic depends largely upon the social and professional qualities of the nurse or attendant. Necessarily the companionship is close and constant, since such a patient cannot safely be left much alone. As a rule, epileptics are not easy persons to manage; the temperament is apt to be unstable, the temper fractious, and the entire disposition variable and *difficile.* Epileptic fits are commonly preceded by periods of irritability and "nerve storm." Usually the patient tends towards mental depression and melancholia. It is an absolute necessity to find bright interests and occupations for epileptics cut off by the nature of their disease from the ordinary social amusements of daily life. The apathy—which so often accompanies epilepsy—makes it a difficult task to provide congenial hobbies and occupations for patients on whose hands time is apt to hang specially dull and heavy.

### Glasses and Games.

Failing, or deficient eyesight, is a common accompaniment of the disease, and this in itself interferes with many pursuits and interests otherwise possible. In some severe cases, the doctor may not think it desirable for glasses to be used constantly, owing to the danger of injury if a patient fall in a fit whilst wearing them. Even when no definite eye trouble exists, any over strain or over use of the sight is apt to give rise to a seizure. So that in finding employment for "idle hands to do," the nurse must bear in mind the necessity for occupation without eye-strain. All open-air games and interests are good, so long as they are not pushed to a fatiguing point. Croquet is an admirable game for epileptics, though I have met with some cases in which the game had to be limited to one hour daily. Many patients complain that croquet causes considerable strain to the eyes and brings on headache. Thus, over-use of the croquet lawn has been found to aggravate and induce fits in some confirmed cases. An important point which needs emphasis is the fact that the midday heat and sun glare of summer is an immediate cause of fits in the epileptic. Epileptic seizures are much more common in summer than in winter. A very great authority on this disease invariably warns his patients to remain indoors between twelve and four P.M. during the hot summer months.

### The Chief Purpose of a Nurse.

It is a great point gained, if the nurse engaged for an epileptic patient is fond of gardening and flower growing. I know of several epileptics in town and city who owe a deep debt of gratitude to a nurse who has interested and instructed them in window gardening. The pleasure attaching to an hour's daily devotion to the growth of box or pot plants, comes as a distinct boon in the aimless lives which their affliction entails on many epileptics. To keep her patient cheerful, occupied and interested, is the chief purpose of a nurse in such cases. This end should be reached without mental excitement or undue bodily tire. The nurse will frequently have to combat a dull apathetic temperament, which rouses to interest for a day or two so long as a pursuit is fresh and novel, and then sinks back to melancholy and depression.

### Sympathetic Co-operation Wanted.

People who have suffered from epilepsy since their childhood are usually neglected from an educational point of view. Commonly they are neither taught to play the piano, to paint, or to draw. In too many cases they have been allowed to drift into listless, uninterested ways, sitting for hours in a state of apathy. And a deficiency in the powers of observation is very noticeable in some epileptics. To draw these patients into the common interests of life calls for the most sympathetic co-operation on the part of a nurse. The mental outlook of so many epileptics is cheerless in the extreme. Girl and women patients suffering from this disease are apt to fall into careless customs as to dress and appearance. Cut off as they are from all but a very restricted social circle, they easily acquire a habit of thinking "it doesn't matter how they look." It is a great point to the good to rouse a wholesome vanity and care for personal appearance in patients who tend to drift into this hopeless mental attitude.

### Clothing and Habits.

A very important point in the personal hygiene of epileptics is that they should not wear tight clothing. A "pulled-in" corset is often responsible for a seizure or a series of seizures; female epileptics aggravate the tendency to fits by putting their hair at night into very tight crimping and curling pins. There are no such things as trifles where epilepsy is concerned. In many cases where the tendency is strong, a very small stimulus will cause a fit. Personal and domestic hygiene is an all-important factor in their care. Healthy regular habits, plenty of fresh air and outdoor exercise, careful dieting, and bright, unexciting interests do much to ward off the recurrence of attacks and to make otherwise dull, dreary lives comparatively cheerful and happy.

### The Fits and Diet.

Cases of epilepsy differ materially. Some patients have their fits in "crops." That is to say, they have a succession of attacks—three, four, or more perhaps within twenty-four hours—after which they may be quite free from fits two or three weeks. In other cases the attacks come singly and very frequently. Some patients tend to have their seizures in the daytime, while in others the attacks are confined almost exclusively to the night. When three to four fits have taken place during the night the patient will usually be considerably exhausted in the morning. The "nerve storm" has been violent, and a re-action of depression almost surely sets in. Fits are sometimes followed by extreme nausea, going on to violent vomiting. This is more especially true of the type of patients whose fits come in "crops." In these cases the dietary must be simple and light. Broths and weak beef teas, small quantities of Valentine's meat juice given in aërated water, peptonised cocoa and milk, are all suited to the nausea and vomiting of an epileptic.

(*To be continued.*)

### Beyond the Seas.

### A NURSE'S LIFE IN SHANGHAI.

The little band of three nurses who, at the request of the Municipal Council of Shanghai, came out here five years ago, were the first private trained nurses to come to China. This, the most English city of the Far East—30 years ago but a mud flat—had come through innumerable illnesses, with Chinese or half-caste nurses, totally untrained: its only general hospital, which was principally for paying patients, being till recently in the hands of a Roman Catholic order. Even now, nursing as may be imagined, is very different here to what it is at home. Domestic servants are plentiful, and people, themselves little above the working class, get used to much personal attendance from "boy" or amah, the servants neither expecting nor meeting with much consideration from their employers.

### Nurses as Machines.

Consequently, many employers of European nurses are still in the initial stage of looking upon them also as a clock, wound up to go on for ever, or at best as a glorified sort of amah. The impossibility of getting help in the shape of a second nurse makes it doubly difficult and the female relatives of the sick people, as a result of climate and the idle Eastern life, are generally useless. Thus a nurse has frequently to go without her proper rest and time off duty. The smallness of the houses and consequent dearth of accommodation add to the hardships. In a Shanghai house belonging to rich people such a thing as a spare bedroom is almost unknown, so that the nurse has to spend all her time in the sick room and snatch what rest she can. Dressing-rooms, as we have them in England—that frequent haven of a nurse—are conspicuous by their absence, most bedrooms only having a bathroom attached.

### The Cost of Living.

Rents are high, and what looks like a large house often contains only two bedrooms and two sitting-rooms, much space being devoted to verandahs, staircases and passages. Living in Shanghai, apart from rent and clothes, is considered cheap. Meat of a good quality ranges from 3d. to 6d. per pound. Vegetables and fruit are to be bought for a few cents per pound. Tea is about half the price, sugar about the same as at home. Enormous quantities of tinned food of all kinds, including butter, are imported and are not much dearer than at home. Bread and milk are, however, higher in price. There is no pasture, so that the cows being fed on bean-cake, their milk is not so nourishing and young infants cannot be fed on it. Vegetables in variety are much the same as at home; of potatoes, peas and beans there are several crops; there are cabbages, cauliflowers, sprouts, bamboo shoots (peculiar to China and very nice) artichokes, carrots, turnips, parsnips, onions, celery, lettuce, mushrooms, spinach, asparagus, but none of them so good as English-grown vegetables. For fruit we have bananas, oranges, mangoes, persimmons, strawberries, cherries, plums, peaches, apricots—the two latter the Chinese pluck before they are ripe; persimmons are much like tomatoes to look at, only sweet, and are very good fruit. There are also pomeloes, in appearance like a much exaggerated orange, which cut in sections make an imposing dessert dish. They have quinine in some way ingrained in their composition and are considered a very safe fruit. This is precisely what melons are not, as the Chinese, in order to get them big and swollen, puncture them and throw them into the germ-breeding creeks, which every village possesses. Melons so treated are hard to detect. In all cases if left cut and standing, they are considered very deadly, owing to their particular attraction for germs, and are a fruitful source of cholera, etc. Game and poultry are plentiful and cheap—the pheasants being very good and from 8d. to 10d. each there are also quails, pigeons, wild duck and geese, partridges, snipe, woodcock, turkeys, all are cheap; chickens are about 3½d. a lb. Domestic duck and goose, like veal and pork, are not recommended, for obvious reasons. Fish is abundant and cheap, but with the exception of samli, a kind of white Chinese salmon, not very good. In the summer months it is not considered safe to eat fish, owing to its rapid decomposition. For all eatables we are considered well off, being regarded with eyes of envy by the residents of the more tropical places, such as the Straits.

### The Difficulty of Clothing.

Clothing at Shanghai is very dear, particularly all fancy things, such as millinery, laces, ribbons, gloves. The foreign stores reckon to make 100 per cent. after paying carriage, so that all who can get their things from home. A dollar (2s.) here in these things goes for the value of 1s. at home. For dressmakers we content ourselves with the Chinese tailor, who is often a tantalising individual. He does not fit badly but in the matter of design is hopelessly at sea. They are also very dishonest about the stuff, and will never give up the pieces over. They are willing, however, to put in any amount of labour in the shape of innumerable tucks, frillings, etc. Lately they have not been so very cheap, a plain dress costing for making anything from 6s. to 10s., but for odd-ments, altering and repairing they are very reasonable.

### Relays of Garments Needed.

Clothes are really to a nurse a very troublesome matter out here. The time-honoured custom of the English nurse to have only one good outdoor private dress would be impossible! One requires relays of upper and under clothing, suitable for all kinds of weather, from hot to temperate and from temperate to extreme cold. This is called a climate of extremes. For extreme heat one wants thin wrappers and transparent muslins; for the extremely cold winter, with one's blood already thinned by the heat, one needs the heaviest under-clothing and the warmest furs; and for the moderate weather much the same as one would wear during an English spring or autumn. The winter is short but sharp, not usually commencing till after Christmas, then generally ice and snow lasting over Chinese New Year, which is usually in February. Then it gets gradually warmer and finer; the spring being usually a very pleasant time till the culminating heat, which is intense, arises. In July and August one just exists, and people who hail from India find it more unbearable here than there. There is, of course, the usual attendant crowd of mosquitoes with the necessity for mosquito nets and houses. One does not go out during the day at all unless forced to do so, and then walking is next to impossible! Five P.M. would be the earliest hour one would go out for pleasure, though some like to get up early and take their exercise, riding or otherwise, at 5 or 6 A.M. One is reduced to the necessity of three baths a day in order to avoid prickly heat. Towards the end of August there is a typhoon, and the very hot weather is at an end. September, October, and right on up to Christmas are lovely months, with bright sunshine during day and cool nights, resembling a very prolonged English autumn.

### Prevalence of Mildew.

This summer, till within the last week, has been the wettest known for twenty-five years. The damp in a season like this permeates everything—boxes, drawers, cupboards—all clothes becoming quickly covered with mildew and spots, particularly shoes and boots. It is at all times difficult to keep clothes from the ravages of damp and moth. In summer all woollen clothing and furs, after being well dried and sunned, are put away in air-tight cases. This makes a lot of work; even carpets have to be taken up and treated. Gloves, of all things, are most trying to keep; fortunately one does not wear them in summer, and so they remain tightly packed in bottles.

### The Victoria Nursing Home.

As I have said, till recently Shanghai has had for its hospital only the institution nursed by Roman Catholic sisters; but some years ago the ratepayers resolved to erect one as a Jubilee memorial to our late Queen. This was completed and opened in April of this year. It is a large, imposing building of four floors. There are wide verandahs running the length of the building on each floor. In front there is quite a large space railed off, with large lawn, where they hope to have tennis next year. At the back of the building there are the servants' quarters, the laundry and kitchens, or "cook-houses" as they are called. The ground floor of the home—by the by, it goes by the name of the "Victoria Nursing Home"—is devoted to waiting-room, matron's office and sitting-room, nurses' sitting and dining-rooms, store room, pantry for use of staff, and serving room where all patients' food is prepared for transmission upstairs. There is a spacious hall and a wide staircase which leads up to the first floor; here are the wards for second-class patients. The Home, contains 24 beds and is principally for paying patients, which is a usual plan abroad. There are only four free beds for suitable cases. For second-class patients the charge is about 5s. 4d. per day, for first-class about 13s. 4d. per day. This does not include medical attendance.

### How the Doctors are Paid.

The doctors' system is very different to what it is at home, every family, and indeed every single person, choosing for themselves a doctor and paying him so much a year whether ill or well. The firms and stores also have doctors whom they pay to attend their employés so that any patients sent in, as they always are, by a doctor to hospital or home, pay just the same. The first floor has a ward of nine beds for men, a ward of five for women, and three small wards, two of which are isolated, with a single bed. At the top of the stairs is a large day-room comfortably fitted up with easy chairs and sofas for the use of convalescent patients. All windows in the wards open on to the verandah, which is also much used by patients. The operating theatre is of suitable size and has all necessary adjuncts, including a top-light. Each floor has bathrooms and lavatories. On the next floor, meant for first-class patients, are a verandah and day-room, two wards of three beds each, for which a smaller sum is asked, and several private wards of only one bed. The dispensary and linen room are on the same floor. Each floor has its Berkefeldt filter—very necessary here. Higher still are the nurses' bedrooms, of which there are five, very cool and pleasant, of a good size and with a fine view. The probationers, who are already commencing to come, have cubicles. I ought to make it clear that the new hospital and the private nursing home belonging to the Municipal Council are one and the same. Some of the nurses are kept for in- and others for out-door work. The charges for private cases are from 10s. 6d. to 13s. 4d. per day.

### The Patients.

The class of cases nursed are largely maternity. Typhoid is prevalent—not usually of a severe type—dysentery, a form of cholera, and all such complaints are very common. Diphtheria and pneumonia occur sometimes, tuberculosis very rarely, scarlet fever and measles occasionally. Small-pox is common among Chinese, who look upon it as a necessary disease of childhood, and inoculate their youngsters that they may get it. All Europeans who come out here are newly vaccinated, and are recommended revaccination at least every five years. One does not often hear of cases among Europeans, so that people evidently get immune, for the Chinese drive about in public rickshas with the rash out on them, and all the time you think you are taking precautions, your cook or your "boy" probably has several cases in his own family at his home to which he repairs every evening. Plague has been almost entirely stamped out. Woosing, which is the port, is also the plague station, and has a plague hospital, happily no longer required. Owing to the rigid precautions taken, and the strict medical examination of every vessel coming into port, the disease has been successfully combated, and with one exception there have been no cases. This is the more creditable, as all the ports around, particularly Hong Kong, are affected.

### No Holidays.

One oddity about nursing in Shanghai is that one never gets away from it, which, it must be owned, would be thought a trifle monotonous by those who know what a wandering life a private nurse at home leads. As the municipality pay for the nurses, they are very jealous of retaining their services, and even when they are not busy, will scarcely ever suffer them to go to outposts. Travelling is enormously expensive, for there are no railways; the only egress is by river and sea. The nearest places one could get to in China would be a two to three days' voyage, and cost from £4 to £6 return. Thus it is not easy to arrange for an annual holiday, of which Japan is the happy hunting ground. It is a beautiful country, and there is so much of all kinds to see. But it costs £9 return to Yokohama, and while in port it would be necessary to stay at one of the hotels of which the charges would be from 10s. to 14s. a day, no small item for a limited purse. So that even given the opportunity a holiday for a nurse in Shanghai is a very difficult luxury to obtain.

### A Hospital Sketch.

I took him in my arms, a little bundle of dirty rags, with big, black, beady eyes peeping out from his wizen face, and I loved him then and there. When he was washed and in his nightgown and wee red jacket, I loved him more, for he looked just the tiny soldier-boy he naturally was, and we called him Tommy Atkins.

Tommy's father was a soldier, but he was in South Africa, and God knows if he even knew that he had such a possession, far away at home. But Tommy Atkins seemed to know all about it, he knew far, far too much, he told you that with his bold bright eyes. It was no use saying "It is not worth while for you to fight Tommy, you will never be a soldier-boy, for your arms are so wee, and your hands are just like star-fishes, although they are so brown. How can they ever beat a drum? And your face, do you think it could ever grow large and round, so that you could blow a bugle? No, mannie, give it up. Besides, mother has gone away and does not want her baby boy, and daddy does not even know." He only waved his tiny sticks of arms passionately in the air, and licked with all his might the spoon with which I was feeding him. Very soon he could not even lick. We squashed a soft baby pillow under his tiny shoulders to help him breathe, and trickled milk, drop by drop, down his wee gurgling dry throat.

Day after day he fought on like that, and we knew he would fight to the bitter bit, for was he not a soldier's baby?

One day I came into the ward quickly, and went over to his cot. Tommy turned his little brown face round as I came, and he smiled. No one could ever forget that smile, it was glorious. I cannot tell you what it taught me, for it spoke straight to my heart, but I know it spoke of sorrow and suffering, crowned with joy—unutterable joy—at last, for when I touched Tommy again, I found that he had gone to be a soldier of Christ.

### The Nurses of St. Saviour's Union Infirmary.

By our Commissioner.

A CHAT WITH THE MATRON AT EAST DULWICH.

There would probably be no difficulty about the nursing question in poor-law infirmaries if all poor-law guardians were as judicious and practicable as the St. Saviour's board, to be known in future as the Southwark Board of Guardians, and the conditions of nursing were always as satisfactory as those which obtain at the commodious and finely-appointed infirmary whose exterior and charming grounds must be familiar to travellers on the Brighton Railway between London Bridge and Tulse Hill. But a particularly pleasing situation, far removed from the dust and din of town, would be of little value if discord, indifference, and inexperience characterised the management of this great South London institution. On the occasion of my visit to Miss Armit, the matron, I had also the pleasure of seeing Dr. Richards, the medical superintendent, who, in fact, was present during a portion of the interview. A walk round a considerable portion of the infirmary, through several of the airy and prettily-decorated wards, a glance at the nurses' quarters, and an inspection of the up-to-date and admirably-planned laundry, enabled me to form some estimate of the extent of the work devolving upon the matron and nursing staff.

"It would be interesting," I said to Miss Armit, whose cheerful sitting-room and adjoining excellently-equipped office, is in telephonic communication with the whole of the wards, "if you could tell me something of the progress of the training school, which, I believe, was founded under your auspices."

### The Evolution of Nursing.

"Yes, there was no school when I came to East Dulwich in 1891 from Bolton Infirmary, of which I was matron. At that time the old system of assistant nurses and ward nurses prevailed. A few of the ward nurses held certificates, and all of the sisters; but the assistant nurses were not trained. Ward nurses and assistant nurses could go when they liked to give a month's notice. So that, I suppose, it may be said that since a new start was made in 1892 I have seen the evolution of nursing here."

"Which has, of course, been gradual?"

"Quite so. The number of probationers has gradually increased since we adopted the three years' system until it has reached the present maximum of 66."

### Candidates for Admission.

"For how long do the probationers come on trial?"

"Two months. If at the end of that time they are found suitable they remain, the two months being included in the three years, as in any ordinary hospital. Applications for admission are as simple as possible. Only one paper has to be signed, and it includes copies of testimonials, the originals of which the selected candidates are required to produce when attending before the board. The questions to be answered are of the usual character, except that a candidate is expected to state whether she has previously been in the employment of the guardians of a union or parish, managers of district schools, sick asylums, or the managers of the Metropolitan Asylums District, and, if so, to give full details, including a statement whether or not she has contracted out of the Poor Law Officers' Superannuation Act of 1896."

### Salary and Hours.

"What is the commencing salary?"

"Ten pounds. The second year it is £14, the third £18. Indoor uniform, consisting of four caps, fourteen linen aprons, three cotton dresses (unmade), six collars, and six pairs of cuffs, is provided. If a probationer, having passed the examination, is promoted to be staff nurse at the end of her third year, she receives £20, rising to £25."

"When do the probationers go on duty?"

"The day duty begins at 7 A.M., and ends at 9 P.M.; the night duty is from 9 P.M. to 9 A.M. We allow three-quarters of an hour for dinner; half an hour, we found, was not enough."

"Do you get any complaints about the off-duty time?"

"No. I think that one reason why our probationers are so contented is the regularity of the off-duty time. Every nurse knows exactly what days she will have off during three months, and that she can count absolutely upon them. I am sure that nurses prefer a longer period of off-duty, and less frequently, to a shorter period and more frequently. Each of our probationers on day duty has two afternoons and one evening off one week, and two afternoons and one half-day another. Junior probationers are also given extra leave for the first few weeks, always the most trying. The senior probationers have every third Sunday from 2 to 10.15 P.M., and other Sundays morning, afternoon, or evening; the junior, morning, afternoon, or evening of every Sunday. Once a month they have leave of absence from 6 A.M. to 10.15 P.M. in lieu of one of the half-days."

"Are the holidays equally regular?"

"Yes; they extend to three weeks, and each nurse knows at least a month before she goes when she is to have her holidays. The night nurses have 24 hours' leave at the end of every month, five hours once a week, and sufficient time on Sunday to attend church morning or evening."

"And the sisters?"

"They also have a whole day off once a month, and three weeks' annual leave; also one afternoon, one evening, and one half-day a week; every third Sunday 2 to 10.15; other Sundays, morning or evening. An hour is allowed the sisters for dinner. All the nurses have breakfast in bed on the days they are off duty, and supper is given them in their rooms on their return at night."

### Infirmary Teaching.

"In such a large institution I conclude that there are a great many operations?"

"About 250 every year. Of course, there are the usual lectures on elementary anatomy, physiology, surgical and medical nursing. The medical superintendent makes it a point to ground the nurses as thoroughly as possible in the principles of aseptic surgery. With so many operations they get plenty of experience, and we are as particular to have things aseptic in the small as in the large. The home sister holds classes in bandaging and bed-making and cooking."

"What about examinations?"

"There is a series of test examinations held from time to time, the object being to ascertain how the probationers are getting on, and to coach them for the final examination which takes place in the third year of the course, a full surgeon of a general hospital being the examiner."

"Then the teaching, you think, compares very well with that of a general hospital?"

"I think that infirmary teaching has special advantages. For example, as there are no dressers, some of the nurses do the smaller dressings, the sisters doing the larger. Also there is very little rough work, owing to the fact that there is a good staff of scrubbers. With a small medical staff the nurses are bound to have more responsibility, and they are therefore more likely to grasp essential points quickly. We are very particular in refusing to certificate the probationers unless they pass the final examination."

"What happens in such a case?"

"They have to leave unless their practical work and conduct have been good. In that event, we are glad to recommend the Board to give them another chance.

"Have you a preference as to the age of admission?"

"We prefer it to be from 21 to 26. Each ward has 30 beds. The probationers start in the female wards, and at the end of three months take day and night duty alternately. We attach great importance to the management of children and their requirements. As you saw, we have separate wards for them."

DULWICH NURSES IN DEMAND.

"Do the nurses often stay on with you after the expiration of their training?"

"Very seldom, because they usually obtain good appointments outside, and they like to widen their experience. Our nurses are in great demand, and I constantly receive letters from other matrons asking if I can supply them from here. Our first probationer to complete her training is now matron of the Wirral Children's Hospital at Birkenhead."

"And some of your assistant matrons have become matrons?"

"During the last seven years five of our assistant matrons have left us to take up the post of matron in important institutions."

"You have two assistant matrons?"

"Yes, and two night superintendents. Exclusive of these, there are twelve sisters. Each sister takes charge of two wards, with an average of 30 beds in each ward, and there is a nurse in every ward at night under the night superintendent, our policy being never to leave a ward without a nurse. The duties of sister, or head nurse, were prescribed under order of the Local Government Board by the Board of Guardians on October 19th, 1893, and have not been altered."

ALL VACANCIES ADVERTISED.

"Have you any difficulty in filling up vacancies for probationers?"

"We often have unsuitable applications, but we can always find nice capable probationers when we want them. Judging by the theoretical work, the type of women who choose to become nurses is improving. We always advertise our vacancies.

"I notice that your Nurses' Home is a part of the administrative block. Do you consider that an advantage?"

"No other arrangement could me made, and I do not know that there are any disadvantages. It is a matter for regret that the nurses do not possess separate bedrooms; but, as far as possible, we put friends or sisters together, and, though two sleep in each room, there is a dividing curtain and plenty of air. The sisters have a large bed-sitting-room in which they can receive their friends when off duty, and they always have a fire in winter."

"Then the general sitting-room is for all the nurses?"

"Yes, and they all have meals in the same dining-room but at different times. There are two libraries, one of scientific books and the other of novels. As to outdoor amusements, they play tennis and croquet in summer, and the garden is the best of any infirmary in London, or I believe elsewhere."

SYMPATHETIC GUARDIANS.

"Are many of the staff cyclists?"

"A considerable number. The guardians lately built us an excellent shed for housing the cycles. They are very good to the nurses, and always willing to do anything they can to promote their comfort."

"Among such a large number of nurses I conclude that there are some who belong to the Royal National Pension Fund?"

"Almost all the sisters and senior nurses are members. I recommend the probationers to take out a policy for a small sum in their third year, and increase it afterwards."

And then, when the medical superintendent had joined in expressing his sense of the consideration shown by the guardians—"They are kind enough to print my lectures," he said—I left St. Saviour's Infirmary pretty fully enlightened as to some of the reasons of its exceptional popularity.

### A Millionaire's Model Ward at Pittsburg.

BY A WANDERING NURSE.

THE general luxuriousness and beautiful equipment of American hospitals has become a proverb. But the children's ward of the homœopathic hospital at Pittsburg, in Pennsylvania, would take first prize even were it possible to arrange a big competition of the model wards of all the hospitals in the world. The most surprising item to a casual visitor is the fact that this ward is equipped with real silver spoons and forks! A millionaire coal and oil king of Pittsburg—which is one of the chief colliery districts of the United States—determined that his city should lead the van so far as the provision of a paradise for sick children is concerned. The central idea of the furniture and fittings of the ward is to reproduce a "bower of roses." Therefore, the walls are of a delicate rose pink, shading into a dado of a deeper roseate tint. All around the ward walls runs an exquisitely painted wild rose, whose briars and delicate buds and blossoms stray about in artistic confusion. The cots are of white enamel, with white and hand-embroidered bedspreads. Turning down one of these displays the charming novelty of rose-pink blankets. Each child has a bed-tray fitted to the cot, and painted in pink roses. A liberal use of lavender bags in the linen chest imparts a lasting scent to sheets and pillow-slips. A little white-enamelled rocking chair is provided for the use of each patient, and the dainty crockery decorated with flowers, tropical birds and scenes of child life would grace a Royal nursery. A low white-enamelled dining table serves for small convalescents proud to sit up to meals in the quaint straight backed white chairs—of Chippendale shape—which are ranged round the table. Dainty white bibs, serviettes, and embroidered table cloths have all been furnished by the local millionaire, who wants the sick children to have "a real good time for once in their lives."

Seeing the loveliness and luxury of it all, the onlooker wonders whether it will not have the effect of making the little rose-bowered patients dissatisfied with the plain surroundings of "home." A white screen with painted wild roses, apparently "all a-growing" is set between each cot, engravings of pretty child subjects hang on the walls, gilded cages, tied up with pink ribbons, hold chirping birds, whose mission is to provide canary concerts when the little ones tire of the music of a miniature ward organ. All the windows are decorated with stained glass, "like a real church" said one of the children, and the glare of the electric light is softened by pink silk shades. A huge velvet pile carpet forms a literal bed of roses for babies and children well enough to leave their cots, and here they crawl and play and "rock" in their little white willow rockers. The bathroom, painted in white and rose-pink, is beautiful enough for cupids to bathe in. Each white-enamelled bath and basin is adorned with large running sprays of the wild rose. Pot plants abound in this temple of tubbing and sweet-scented pink soap is always used. To be warded in the Homœopathic Hospital leaves a memory in the minds of all Pittsburg children who have been there of a trip to fairy land. Travellers and globe-trotters are advised to go and see the ward for themselves. It is a unique bit of hospital scenery.

### Echoes from the Outside World.

AN OPEN LETTER TO A HOSPITAL NURSE.

It is a fact probably known to all of you that the first introduction of the Finsen Lamp into England was entirely due to the good offices of our present Queen, then Princess of Wales. And apparently England is not the only country where the love of royal ladies for their afflicted subjects has resulted in untold benefits being brought to them across the sea. A pretty story is told of the Queen of Portugal. Even as a girl she was much interested in all medical and nursing questions, and devoted both time and thought to them. Had she been in another position she would probably have embraced one of the two professions which would have enabled her to minister to the sick. As, however, such a career was necessarily denied to her, she determined to do her best in that state of life to which she had been called. Even as Duchess of Braganza, she set to work to improve the condition of the hospitals of Lisbon, which were in such a disgraceful state that many of the poor, however ill they might be, refused to go into them. Then, later on, after she became Queen, the discovery of the marvellous power of the Roentgen rays was made and owing to her influence the medical authorities of the Portuguese capital agreed to establish the apparatus in the hospitals there. But this was only one step in the right direction, for the Queen, well knowing the prejudice of the poorer people against any new method of which they knew nothing, insisted on being one of the first to be put under the influence of the wonderful light although not, apparently, medically, and caused the fact to be widely published throughout the country that all might know the new method possessed no subtle terror, nor strange danger to life. The account of such a deed of kindly thought for her people, is not the less welcome because the Queen of Portugal is always spoken of as one of the best dressed women in Europe and there are folks who maintain that philanthropy of necessity accompanies dowdiness. It is pleasant to know the reverse.

The second of the five races to be sailed between the English and American yachts—the victor of necessity winning three—took place on Tuesday, but owing to the want of wind was not finished within the limit of time, and will have to be sailed again. *Shamrock* was leading when the signal came "No Race." The first race, owing to the want of wind on Thursday, had to be sailed over again on Saturday. The excitement must have been intense, for it was a neck-to-neck contest nearly all the way, and, although the *Columbia* won in the end, it was by less than two minutes, time allowance included. This was the more notable because there was a brisk wind, which was not supposed by some to be favourable to the English vessel. The defeat was so near a victory that it should go far to soften Sir Thomas Lipton's disappointment. An accident which might have been very disastrous nearly marred the pleasure of the occasion. The *Erin* was run into by another ship, and so serious did the collision appear at first that the captain ordered the boats to be manned. Fortunately, however, the damage to the vessel was found to be trifling, though a couple of lady visitors had been knocked down and badly bruised.

One has grown to believe that except in penny novelettes the days for brigands running off with ladies and then demanding enormous sums for their ransom have long since ceased. But events in Turkey do not bear out the belief. Miss Stone, an American missionary, has recently been carried off by brigands to their mountain fastnesses, and the thieves have sent word to the American Board of Missions that they refuse to give up their captive unless a ransom be paid for her. This the Board have consented to do, but their consternation must have been great to learn that the lowest sum which will be accepted is £25,000 sterling. The poor lady has sent a letter to the American missionary at Samakov to say that she is well fed and respectfully treated, but suffers much from the daily and hurried change of locality, the brigands shifting their halting-place sometimes in the dead of the night so as to avoid any risk of being traced.

The Cat Club held a most interesting championship show last week at the Leopold Institute, Slough, in aid of Princess Christian's Trained Nurses, which was attended by an enormous number of people, with the result that a goodly sum of money was added to the funds of a most useful institution. Amongst the exhibitors were Princess Victoria of Schleswig-Holstein, who sent three cats. The first was called Blue Girl, and was a blue Persian, which carried off first class honours. The other two were Puck III. and Imp II., both beautiful little animals, and one not only won the prize in its class but a silver medal offered by the Silver Society. Lady Aberdeen gave a solid silver model of a cat for the best silver tabby bred by an exhibitor, and it was secured by Lady Pink. There were several Siamese cats in the show.

When Mr. W. W. Astor writes a cheque for a charitable institution it seems to run into five figures as a matter of course. His last act of munificence will cause joy and gladness to the friends of oppressed and unkindly treated children all over the country. It is true thal he has sent the National Society for the Prevention of Cruelty to Children his cheque for £10,000, for the express purpose of enabling Lord Ancaster and the Committee to open a fund for the provision of premises suitable for its requirements; but the more largely the Society looms in the public eye, the more support it is likely to have. With offices worthy of its high aim, and its increasing labour, it cannot fail to command augumented influence, and to exercise increasingly powerful intervention in the interests of child life.

Matrons and others to whose lot it falls to engage domestic servants must be naturally interested in the character question. They are lucky indeed if they have never been the victim of a recommendation forged in some clever manner. One of the most noteworthy cases which I have personally known is that of a cook whose mistress dismissed her for bad behaviour just before the autumn holidays, warning her that, of course, she had forfeited her reference. The lady went abroad and heard no more of the matter, but it has lately come to her knowledge that during her absence the girl applied for a situation, and, when she found a personal character was required, stated that the lady was at home and would be glad to give it on a stated morning. The visitor called and was received in the drawing-room by a tall, well-dressed lady, who in good English spoke most satisfactorily of her late cook. The parlourmaid had kindly dressed up in her mistress's clothes in order to assist her fellow-servant. At the Lambeth County Court, last week, a German lady's maid, who was a witness in a case, produced a book in which all her previous mistresses had written references, each being officially stamped. In reply to Judge Emden she stated this was an almost general custom in Germany. The judge seemed much pleased with the idea, "as it appeared to him to afford protection both to the employer and the employée." But, alas! a lady signing herself "Hausfrau" immediately wrote to the papers saying that last winter, whilst in Germany, she engaged a servant with the usual little green book of first-class references, each duly stamped by the police. This, however, did not prevent the maid decamping one day with all she could lay her hands on, and upon reference to the police-court, no trace of a person answering to her description could be found in any of the bulky volumes lining the official shelves. So that the remedy for false characters has evidently yet to be found.

### Examination Questions for Nurses.

OPENING OF THE AUTUMN SESSION.

I hope that all nurses who intend to compete in our examinations this year are quite determined to do their very best.

Some of our candidates' work is very satisfactory, and looking back three years a very decided improvement may be marked in the general tone of the papers; yet we must not be content with this but still strive for better results.

Those climbing the slippery hillside of knowledge will find standing still an impossibility; we must advance, or a retrograde movement is only too easy.

Once more let me beg you to keep to the question under discussion; a tendency to rush off on loop lines marks the ill-informed. Above all things avoid showing off supposed learning. Do not use Latin words when English will do just as well, the simpler a nurse's language the better. You little know the amusement such pretensions to medical knowledge and terms, cause to doctors.

Another ignorant and vulgar fault I am sorry to say I have noted, though fortunately not often—namely a conceited tendency to criticise the treatment of cases by various doctors, especially if they are what these complacent young women call "country practitioners."

Never forget for one moment that the province of nurses is to carry out intelligently the orders of the doctor—not to treat the case themselves, except in exceptional circumstances, such as I indicate in this month's question.

About two years ago some papers were set relating to the same subject, but, owing to a slight error, they were misplaced, so that it would be well to repeat them. Note particularly the wording and intention of the question, and do not go beyond it.

There is still lamentable untidiness and carelessness shown in the way of writing and sending in competing papers. An untidy scribe will in all probability be an untidy nurse. In these days of cheap paper and envelopes, it is surely unnecessary to write on torn sheets from ledgers, and to post the same without an envelope and feebly tied with thread. These trifles mark the incurable sloven, a person to be carefully avoided in the sick room.

The Question for October.

Supposing yourself called to nurse a case of severe typhoid fever without medical direction, how would you feed the patient whilst liquid nourishment alone was permissible?

The Examiner.

### Appointments.

Exmouth Hospital. — Miss Jane Jeffery has been appointed nurse-matron. She was trained at the Royal Hospital for Sick Children, Edinburgh, at the Middlesex Hospital, and at the Battersea branch of the Clapham School for Midwives. She has since been head nurse at Newton Abbot Workhouse Infirmary, and head nurse at Cheltenham Workhouse Infirmary. Miss Jeffery holds the L.O.S. certificate.

Memorial Hospital, Kendal.—Miss Evelyn A. Mansel has been appointed matron. She was trained at Charing Cross and Guy's Hospital, London, and has since been matron at Easingwold and Ashley-de-la-Zouche Hospitals, assistant matron at the Royal Hospital for Sick Children, Edinburgh, and holiday sister at St. George's Hospital, London.

Middlesborough Union Infirmary. — Miss Ada F. Rockett has been appointed superintendent nurse. She was trained at Leeds General Infirmary for three years, and at Queen Charlotte's Hospital, London. She has since been assistant matron at the British Lying-in Hospital, London; superintendent nurse at Hunslet Union Infirmary, and is at present temporary surgical sister at Derby Royal Infirmary. Miss Rockett holds the L.O.S. certificate.

National Hospital for Consumption, co. Wicklow, Ireland.—Miss Josephine Molony has been appointed sister. She was trained at the Poplar and Stepney Sick Asylum, London, and the Northern Branch of the Hospital for Women, Brighton, and has been for some months staff nurse at the Bromley Cottage Hospital, Kent. Miss Molony holds the L.O.S. certificate.

Princess Mary Convalescent Home, Bognor (sea-side branch of East London Hospital for Children),—Miss Lucie Havers has been appointed superintendent nurse. She was trained at the Jenny Lind Infirmary, Norwich; the Children's Hospital, Glasgow; and the Leicester General Infirmary. She has since been sister, for seven years, at Leicester General Infirmary, and matron at the Cotswold Sanatorium.

Totnes Union.—Miss Louisa Foley has been appointed assistant nurse. She was trained by the Meath Workhouse Nursing Association at the Mildmay Memorial Hospital, Mildmay Park.

Wakefield Union Infirmary.—Miss Hilda Mary Hannah Barnacle has been appointed sister. She was trained at Leeds Union Infirmary.

Willesden Isolation Hospital.—Miss Maud Cooper and Miss May Littleboy have been appointed ward sisters. They were trained for three years in the same institution.

### "The Better Land."

By A Pessimistic Nurse.

With apologies to the late Mrs. Hemans.

I hear thee speak of a better land,
Where the hours are short, and the pay is grand,
And the mess-room table holds sweets galore,
And the Matron gives from an ample store;
Where every Pro. wears a smiling face,
And every op.'s a successful case,
And the "Rule-Book" all can understand!
Oh! tell us, where is that better land?
"Not here in London, my child!"

A land where the maids work willingly,
And patients recover, and seldom die,
And both "diet-lists" and "stock" come right,
And "Committee Inspectors" come not in sight,
Where Office, and Stores, and Dispensary,
Whatever is ordered *at once* supply;
And all turns out as you've duly planned!
Oh! tell us, where is that better land?
"Not here, not here, my child!"

A land where the uniform's fit to wear,
And the ward-provisions you needn't spare,
Where the doctors behave like angels all,
And the surgeons are never upset at all!
And "theatre-passes" a frequency,
And mutton at table a novelty,
And up-to-date magazines always at hand,
Oh! tell us, where is that better land?
"Not here, not here, my child!"

"My gentle child, where's your common sense?
Such a place may be found just a century hence!
Sorry am I to give you pain,
And tell you your castle exists in Spain!
In mental visions alone it lies,
A fairy palace in dreamland skies;
There's never on earth such a land at all!
It's *Heaven* you're wanting, not Hospital!
So just be content, my child!"

### Everybody's Opinion.

[Correspondence on all subjects is invited, but we cannot in any way be responsible for the opinions expressed by our correspondents. No communication can be entertained if the name and address of the correspondent is not given, as a guarantee of good faith but not necessarily for publication, or unless one side of the paper only is written on.]

### 114 APPLICATIONS FOR A POST OF MATRON.

"B." writes: 114 applications for the post of matron! A goodly number, certainly, but not a record: for at the time the present matron of the Bridgenorth and South Shropshire Infirmary was elected there were no less than 125 candidates for the appointment.

### NURSES AND THE CORONATION.

"A Pension Fund Nurse" writes: I have been wondering whether by any means you could help nurses who would pay a moderate sum to get a sight of the Coronation procession. Nobody would appreciate the sight more than the Royal National Pension Fund nurses, and I know no one who would be so likely to help as yourself. If you could hear of any arrangement by which nurses could be accommodated perhaps you would let us learn of it through The Hospital, as I am acquainted with many who would be glad to subscribe in advance.

### NURSING IN WORKHOUSES.

"Trained Nurse and a Scotswoman" writes: Replying to "Scotswoman," who wrote in your valuable paper a fortnight ago, may I be allowed to say that, while advocating trained nursing for the sick pauper, after over a year's work in a parochial hospital, I consider something more is due to the nurse who takes up the duty. I never heard such awful language as I have heard here, though I was working for several years in the East End of London. The domestic arrangements, to say the least of it, are very indifferent, and, as for the uniform, well, when I say that I wore one serge dress for a year in a ward where the habits of the patients are none too clean, its condition may better be imagined than described. These may seem minor matters, but when the long hours that nurses are on duty in poor-law institutions, the class of people they have to be with, and the monotony of the work are taken into consideration, you will, I think, agree with me that until some little reformation is brought about the parochial authorities may certainly get trained nurses, but will find it a difficult matter to keep them.

### TWO YEARS' TRAINING.

"M. O." writes: I noticed this advertisement in a daily paper:

WANTED,—Young women of good character and education (none under 25 years of age or over 30 can be received), to be trained at a hospital for two years, and afterwards to be employed as nurses for the sick in private families; entrance fee £1.—Apply Lady Supt., &c.

Surely it is some years since two years' training were deemed sufficient for a nurse. One is led to suppose from this that many of the private nurses one works among have simply had two years' training in some hospital, undergone no examination, and own no certificate. I consider this most unfair to those who have gone through a proper training of not less than three years, have passed examinations, and thus gained their certificates. We are all, of course, classed as private nurses. Will you kindly give your opinion of the advertisement? Or is it too old a grievance?

[We are afraid that a good many nurses of insufficient training are sent out by minor private institutions. This will continue until the public have the wisdom to insist upon seeing the nurse's credentials.—Ed. The Hospital.]

### TRAINED NURSES AND HOUSEHOLD DUTIES.

"Cricket" writes: In a case of tracheotomy or bad diphtheria do you consider a nurse justified in undertaking to clean the fireplace of the sick room? Personally I do not; as, although it may only take a few moments to pull off dusty apron and gloves, every nurse knows the remarkable tendency of patients to be taken worse at the very minute one's attention is divided; and in a case so critical promptitude and cleanliness may just alter the balance between life or death. I do not recommend young servants who attend to the rest of the household coming into the sick room, but a woman of the charing class is usually to be found who is willing to come in each morning for that purpose, the work being done in a very short time by one who is accustomed to it, her coming and going through the open air giving no more risks of spreading infection than the doctor's visits, as she does not go near the patient. That is what I advise to patients' friends, and have always found them willing to carry it out and anxious to spare no expense for their sick one's good. The nurse is then free to give all her attention to the work in hand: it is surely due to her that she should be, as she has the responsibility. I mention this because some have thought me too particular, and I would be glad to have your opinion.

[If the grate really has to be cleaned during such an acute stage of the disease, evidently some one must be found to clean it.—Ed. The Hospital.]

### THE CURETON TESTIMONIAL FUND.

"Peggie" writes from 2 Rue d'Aguesseau, Faubourg. St. Honoré, Paris: Can anyone give me some information about the above fund? I am an old Cambridge nurse, and had the privilege of Miss Cureton's friendship all the time I was at Addenbrooke's Hospital. Indeed, all the nurses looked on her as a friend. At the same time she was a perfect matron, a combination in my experience not often found. Some time since I received a printed form from Miss West on the above subject containing a number of names, none of whom were familiar to me, which is not to be wondered at, as I have been nursing in Paris for seven years, also in London, etc., since I trained at Addenbrooke's. So I sent my offering to Barclay's Bank. In due course I had an acknowledgment of my cheque from them, but they evidently knew nothing of the fund, and on reference to the papers from Miss West I found it was to members of committee, whose names were given, it was to be sent to, and they would pay it into Barclay's Bank. I enclosed the paper to the manager of the bank, asking him if he would kindly put matters right for me. Since then I have heard nothing, and shall feel obliged if anyone through your columns can give me any information on the subject.

### EXPERIENCE OF A CERTIFICATED PRIVATE NURSE.

"E. L." writes: I agreed to take the entire charge, for three months, of a first baby seven months old. The responsibility of the child's health and mode of feeding was to rest with me, for its mother (who was delicate) did not wish to have the worry of the baby's upbringing, as it was likely to have rickets within the next eighteen months. A house was engaged by the infant's grandmother in the country, where we were going on a visit for several weeks; and after receiving written instructions from the medical man who attended at the child's birth, I went to fill my post. On reaching the house I found that I was expected to sleep with the child in its mother's bedroom. As I did not think this arrangement very satisfactory I suggested that I should take the child and have a room for her and myself. This the mother eventually consented to, and I was allotted a small room in which to sleep with the infant. This was not the only difficulty which cropped up. Contrary to the directions which had been given me before leaving London, the grandmother and aunt of the child each suggested various diets which they thought would improve it. Now, as I was to be responsible for the health of my charge, I thought that the doctor's instructions should be obeyed, and not two or three different modes of feeding, as the child improved in weight each week and was doing well. My life was not a happy one, as I was very much worried by the injudicious interference of the child's mother and relatives, when I had been engaged as a thoroughly competent nurse to look after the child on my own responsibility, and refused other work to do so. At last the climax was reached by having the child taken from me about 8 A.M. one morning (a month after my arrival) by its grandmother, who, without consulting me, took her for the day on a steam yacht on the sea with the family, and I was ordered to bring its breakfast. I did so, and told the grandmother that I could not be responsible for the child's health if it were taken from me in that manner for the day, with no one to prepare its food. In consequence of this act of interference on the grandmother's part, I was compelled to give up my post. Would you kindly tell me if I am entitled to my salary up to the end of the three months for which I was engaged, as I ask in the interest of many of your readers who may perhaps have similar posts offered them?

[If, as we gather, you voluntarily threw up the post, you are, notwithstanding the provocation you received, only entitled to payment for the time you had charge of the child. Ed. The Hospital.]

### THE SCARCITY OF NURSES AT THE WORKHOUSE INFIRMARIES.

"A Poor-law Nurse" writes: I have been much interested in your remarks about the "Scarcity of Nurses at the Workhouse Infirmaries." As a poor-law nurse may I venture to give a few reasons for this and to suggest what, in my opinion, would be a cure for this dearth? A chief reason is that the wrong woman is chosen by the guardians. As a rule the poor-law nurse has no pretension to being a lady, or even to being ladylike. Fast, flighty, drunken, lazy, ignorant, and underbred, some of these are adjectives which may, alas! with truth be applied to the present poor-law nurse, with only a few exceptions, in most of the infirmaries, although in some a better tone prevails. This criticism applies not only to the probationers and recently-appointed nurses, but to all the nursing staff from the matrons downwards. What inducement has a good nurse to go amongst such women, to associate with them daily, to be under their orders, and choose from among them her friends? Another reason is that the training is poor, and that, although there is good work, the sisters do not teach the nurses, and in many cases they are unable to do so. They are not particular enough; faults are glossed over; the "tone" is not what it should be; there is no *esprit de corps*—no desire to work to make the thing go, but merely to do as little as possible in the day. In a large number of cases it depends upon the nurses' own efforts whether they get trained or not. The third reason is that very many infirmaries do not profess to be training schools, nor even attempt to be so. There is no reason why the training should not be the best in the world, because in the absence of students the nurses get far more real "nursing" than in the general hospitals. In the latter, too, the sisters do a great deal of the nursing which in infirmaries is given to nurses. Moreover, the midwifery might be made of more use than it is at present in teaching the poor-law nurse, and the opportunities for taking the L.O.S. might be made such as would not occur to the nurse in a general hospital. The remedies to combat these evils, in my opinion, are as follows:—(1) Get a good class of nurses, sisters, and matrons, respectable, sober, honest, bright, and intelligent women who love nursing, and who have, if not the birth, at least the feelings of a gentlewoman; (2) make the sisters, matrons, and doctors give a good practical and theoretical training to the nurses; (3) and this follows on the last—let the infirmaries profess to be good training schools, and give a certificate worth having. The nurses will then feel proud of a certificate which they have gained by conscious merit, and will be only too proud to stay in the hospitals and help to teach others.

### For Reading to the Sick.

### DIVINE REST.

"*Come unto Me . . . and I will give you rest.*"

Between the mysteries of death and life
Thou standest: loving, guiding, not explaining,
We ask and Thou art silent: yet we gaze,
And our charmed hearts forget their drear complaining.
No crushing fate, no stony destiny,
Thou Lamb that hast been slain, we rest in Thee.

. . . . . . . .

Thy pierced Hand guides the mysterious wheel,
Thy thorn-crowned brow now wears the crown of power,
And when the dark enigma presseth sore,
Thy patient voice saith, "Watch with Me one hour."
As sinks the moaning river in the sea,
In silver peace, so sinks my soul in Thee.

*Harriet Beecher Stowe.*

Lord since our griefs on Thee were laid,
And Thou has felt their sting,
Help us in holiest calm to take
Our turn of suffering;
Thou didst look on unto Thy joy,
And so by grace will we,
But we would clasp Thy Cross and feel
We owe that joy to Thee.—*C. M. Noel.*

Man's nature needs a higher nature than its own to be its stay, its peace. A higher Presence must enter within it, to become part of itself, or there is a void, a loneliness.

*T. T. Carter.*

Not by removing burdens, but by giving a spirit and a power to bear the burden; not by changing thorns into roses, but by helping us to bear the thorns; not by leaving us alone, but above all, in and through all, by the power of felt association with Himself—by the union of our Baptism, by the perpetually renewed oneness of the Holy Communion—Christ gives us rest here and now.

We so tired, so restless, so weary, utter the old moan of impatience, "Oh, that I had the wings of a dove! Then would I flee away, and be at rest." But by degrees we feel that no wings would help us, no mere escape would give us what we want. It is not the wilderness that we really want; it is love here and now, it is interest, it is companionship, it is relief from our loneliness, it is the Hand pierced for love of us, it is the contact with a heart that can feel with us, that knows and understands and can take us in, that at once warns us and encourages us, and that points out the road—the path of duty, the path of service, the path of active self-sacrifice, and then gives a motive, "for my sake," and says, Here is your rest: "Whosoever will save his life"—by keeping it from trouble—"shall lose it; whosoever will lose his life"—fling it away—"for My sake shall keep it unto life eternal." And we must let Him teach us, else we shall be restless to the end. We shall look on to death to deliver us, but we shall be more restless in death, when left by ourselves and to ourselves, we shall go on to the end, wailing, "Oh, that I had the wings of a dove! then would I flee away," and never learn the truth—that, not in fleeing away, not in the wilderness, not in happiness, not in enjoyment, but in taking the yoke and bearing the burden, in fellowship with love itself, there and there alone we shall find "rest for our souls." And that because there we shall find peace within, repose in an unseen Presence, on an unfailing Love. The surface of the ocean may be broken by waves, lashed by winds, storm-tossed, restless, but beneath is the unbroken repose of the depths of the sea that no storm affects and no waves disturb.—*Robt. Eyton.*

### A Book and its Story.

### HISTORY AND ROMANCE.*

The most prosaic person cannot fail to find in the latest addition to historical romantic literature, under the title of "Secret Chambers and Hiding Places," a book of unusual interest from the thrilling stories it contains of devout and persecuted persons who found sanctuary in the grim "holes" and hiding places still existing in many of the manor houses and old halls of Great Britain. From remote times the necessity for such places has existed, and after the Reformation, and following on Gunpowder Plot, the sanguinary laws against priests and those who refused to take the Oath of Supremacy were enforced with greater severity. No member of the Church of Rome could celebrate the rites of his religion without risking terms of imprisonment or forfeiture. In those days, as Lord Tennyson wrote, "What's up is faith, what's down is heresy," and places of safe retreat for those who were victims of the party in power became a necessity.

The mansions of old Roman Catholic families had nearly all their secret apartment hidden away in the roof, in a distant part of the house, or in some remote garret where, undisturbed, they could worship in peace. Adjacent was an artfully contrived hiding place for the priest in case of emergency. The sacred vessels and vestments, the altar furniture, and every trace of anything likely to excite suspicion was also stowed away with him. Many of the hiding places called "priest's holes" were constructed by the ingenuity and inexhaustible energy of a diminutive Jesuit to whose shortness of stature was added enormous strength. He was the faithful servant of Father Garnet, and a contemporary writer speaks of "his being able with incomparable skill to conduct priests to a place of safety along subterranean passages, to hide them between walls and bury them in impenetrable recesses, and to entangle them in labyrinths and a thousand windings." These retreats, of which "Little John" was the sole architect and builder, were often constructed between walls, which had first to be broken into and excavated, the huge stones which were displaced requiring no ordinary strength for their removal. But "Little John" worked valiantly at his labour of love, and so cunningly were the designs carried out that the entrance to the hiding place was concealed in such a way that no suggestion of its existence could be found in its vicinity. "It was not an uncommon thing for a rigid search to last a fortnight. Skilled carpenters and masons would try every possible expedient, from systematic measurements and soundings, to bodily tearing up the floors, and in the end they would go away empty-handed, while the object of their search lay concealed within a wall's thickness of his pursuers, half starved, cramped and sore with prolonged confinement and almost afraid to breathe lest the least sound should throw suspicion upon the spot in which he was immured." Many hunted co-religionists owed their lives to Little John's skill in the troubled days which succeeded the Reformation. Hindlip Hall is specially spoken of as a mansion in which more than one of his masterpieces was constructed. Its owner was a strong partisan of the persecuted Mary Queen of Scots. For his devotion to her and his avowed allegiance to the Church of Rome he suffered a long term of imprisonment in the Tower. On being set at liberty he at once engaged the services of Little John to assist in forming a harbour of refuge for those of like sympathies under his own roof. "The walls of the mansion were literally riddled with secret chambers and passages. There was little fear of being run to earth with hidden exits everywhere. Wainscotting, solid brickwork or stone hearth were equally accommodating and would swallow up fugitives wholesale, and close over them, to 'Open Sesame!' again at the hider's pleasure." Not less closely concealed in the ingenious brain of "Little John" was the locality of the various hiding-places, and so loyal was he to his church, that when he was arrested with his master, Father Garnet, by the order of Cecil, it was directed "that first the secret was to be coaxed from him, if he be willing to contract for his life," but if not, "it is to be wrung from him." Not even the horrors of the rack could extort one word that would have given into his accusers' hands the lives of those for whom he had spent his days, and he died under its torture rather than make any revelation. The chief attraction of these records consists in their authenticity, for they are taken from contemporary writings, otherwise the thrilling narratives of hairbreadth escapes and of personal heroism and endurance, would be almost too wonderful to be true. Here is one taken from "The History of a Great English House." It has reference to the tragic fate that overtook Francis, last Viscount Lovell in a secret vault beneath the Manor House of Minster Lovell—the ancient seat of the family. Having sided with the cause of Simnel against Henry VII., he fled after the battle of Stoke to his mansion in disguise. From that night he was never seen or heard of again, and for nearly two centuries his disappearance remained a profound mystery. The Manor House in the meantime had fallen on hard times and the greater part of its stately walls were allowed to fall into ruin. In the year 1708 in the remaining portion, tenanted by a farmer, the stone chamber beneath was discovered. Here, seated before a table with a prayer-book lying open upon it, was the entire skeleton of a man. Barrels and jars were found which had contained food sufficient to last for some weeks. The mansion was seized by King Henry shortly after Lord Lovell's disappearance, and it is concluded that, unable to regain his liberty through the neglect of those to whom his hiding place was known, he met with his tragic end. When we realise the hardships endured by our ancestors for loyalty and conscience' sake, and the confined space in which they dragged out their weary waiting, not always ending in freedom, we can but be thankful that we live in more peaceful times. In choosing secret chambers and hiding places in the ancient mansions as a subject for research Mr. Fea has chosen one inexhaustible in its interest and of more tangible nature than that of the family ghost, who, "since the searchlight of science has penetrated into his sacred haunts has no longer a leg to stand upon." But the secret chamber has its special interest for the antiquarian mind, and therefore holds a dignified position in its own department. The numerous illustrations of these curious corners above and below, with the mode of entrance to their secret recesses, and also, of the beautiful old mansions and castles of which they form a part, enhances the value of this very interesting volume.

* "Secret Chambers and Hiding Places." By Allan Fea. Illustrated. (Publishers: Bousfield and Co., London. Price 10s. 6d.)

### Wants and Workers.

Will any kindly disposed ladies having any cast-off clothing (infants' or small children's especially), no matter how old, allow District Nurse, Cawston Lodge, Haverland Park, Norwich, to have them for free distribution to the poor amongst whom she works? There is a real need and few to help in the several parishes.

### Novelties for Nurses.

By Our Shopping Correspondent.

### DOMEN BELTS.

"Support without Pressure" is the watchword of the Domen Belts Company, 456 Strand, W.C., and the latest development is a combined straight-fronted corset and belt, which there is no doubt women anxious for an elegant figure without tight-lacing will appreciate. This straight-fronted form, it is claimed, will be found of great benefit and assistance, while the absence of compression in the epigastric and umbilical region cannot fail to meet with the appreciation of sufferers from gastric troubles. This belt corset is really a combination of a corset with a belt, and has all the advantages of each without the inconvenience entailed when the separate articles are worn. It possesses one great superiority over the ordinary simple corset, that it prevents the down pressure on the internal organs, which even moderately tight lacing involves; while it gives efficient and valuable support both to the spine and to the abdomen.

Close attention has been given to the production of special belts for special complaints; and nurses will be glad to know that the manufactures of the Domen Company are a scientific improvement on the old-fashioned notion that a belt useful in one case must be equally so in another.

Thus, a special belt is made for accouchement and corpulency; while the hypogastric belt is used for extreme cases; its action is most beneficial when the womb is pressed up against the bladder, and when the patient suffers from the pains which so frequently make themselves felt in the hips loins, and sides.

The construction of the "Abdominal Support" with spinal springs is peculiarly adapted for relieving pains in the back, and disorders caused by weakness of the spine. Attached to the ends of the support are two covered flexible cushioned pads, which act as stays to the spine. In extreme cases of stoutness, the padded springs prevent the support from cutting the body or pressing upon the spine.

A special support is made for umbilical hernia. In the case of an unusually stout person suffering from umbilical hernia, and especially with women past middle age, who have borne large families, the abdomen, from its relaxed condition, has a tendency to push an umbilical spring truss out of position. In such instances the excellent shape and construction of this belt maintain it in position, affording an exceedingly comfortable stay to the abdomen and the internal abdominal organs, without requiring it to be drawn too tight.

The Domen stoop cure is constructed of an anatomically shaped back-piece, containing watch-spring steels running parallel with the spine, and two springs extending from the centre line to the shoulders, with straps which draw the shoulders backwards, and thus increase the size of the thoracic cavity. The Domen Company's specialities are used in many of the leading hospitals; they may all be seen on the first floor of No. 456 Strand, where their several purposes are explained by the manageress.

---

### MESSRS. EGERTON BURNETT'S DRESS MATERIALS.

In the large box of patterns received from Egerton Burnett, Limited, of Wellington, Somerset, there is as usual a great variety of choice in price and quality. There are pretty heather mixtures for coats and skirts for out-of-uniform wear, bright blues, greens, and reds for people of a cheerful turn of mind, and more sombre blacks and navy blues or greys and browns for those who want to make their dresses last and not show the dirt of winter streets. By the way, for any nurse who is really going to be bold enough to drop the skirt altogether and appear in "rational" dress let me recommend a handsome tweed, or a manly "coating." The "Cornwall," for example, would look very neat; there are two shades, a grey and a mouse-coloured brown. If rational dress is to be worn it should be made in quiet colours, unless one carries out the old saying that it is as well to be hung for a sheep as a lamb, in which case it does not much matter what colour one chooses in which to run the gauntlet of public criticism. However that may be these charming materials would make up well in a "rational" or any other style.

A very nice soft cloth is called the Marguerite; it is wide—45 inches—and the price is 2s. 9d. There is a great choice of colour, but perhaps the neatest for skirts is a navy blue; it would do also for uniform cloaks, as it is not at all heavy, though warm. The "Hursley" is another useful material, very suitable for cycling, and the "Pyrmont" is pretty, though this, I think, would look nicer without the white hairs; however, it is fashionable to have them, even though they may sometimes suggest that one has been nursing the cat. The patterns mentioned by no means exhaust those in the box; but it is time I said something about blouses, and for these there are fancy flannelettes, one at the extremely moderate price of 6d. a yard, width 27 inches. Black, with narrow red stripes, blue or black with white stripes, red with black stripes, or blue or black with a small pattern—either would be serviceable, and look comfortable for the winter. For a slightly higher price one can have a larger choice and brighter colours, in Paisley patterns or checks. There are tartans, too, at 2s. 6½d., width 41 inches, suitable for dresses or blouses, and there is a pretty silk for about the same price.

The huge leather-covered samples of washing materials for nurses' uniform dresses, and the waterproof cloaking which is so serviceable to those who have to brave all weathers, next claim attention. The strong galateas made by this firm are always in demand; here is one for 6¾d., 28 inches wide; it is a twill, with narrow blue and white diagonal lines, and feels strong and durable to the touch. The "Nightingale Stripe" is another very neat material, 10¾d. a yard, a speciality for nurses' washing dresses. It is made in blue, black, and red, each with narrow white lines running through.

For "Probationers' Aprons" there is a strong white cotton at 9½d., wide width; and the hollands, too, are very cheap and lasting. Nainsook, cap muslin, and dress linings follow; and then come the serges, made with pure wool and permanent dye, in navy blue, white (washing serge), and black; and then the waterproof cloakings referred to above. Cloaks are made to order if particulars are sent to Wellington, and I need hardly remind my readers that for really serviceable cloaks these materials are among the best. I may add that Queen Alexandra and the Empress of Russia have recently sent orders to Messrs. Egerton Burnett for dress materials.

---

### AN EXCELLENT WOOLLEN FABRIC.

Probably most of the readers of The Hospital are familiar with an excellent woollen material called Orlwoola, but if there are any who are not, I would call their attention to it. It is manufactured for all the purposes to which a beautifully soft, fine, and light woollen material can be applied. Being unshrinkable, it is especially suitable for underwear, but the designs are so tasteful that it provides charming dresses for children, blouses for adults, tennis dresses, and dressing-gowns. It is an ideal fabric for gentlemen's pyjamas and for athletic purposes. It is manufactured by the Leigh Mills Company, Coventry, and is sold by all drapers of good standing.

### Notes and Queries.

**The Editor is always willing to answer in this column, without any fee, all reasonable questions, as soon as possible.**

**But the following rules must be carefully observed :—**

1. **Every communication must be accompanied by the name and address of the writer.**
2. **The question must always bear upon nursing, directly or indirectly.**

**If an answer is required by letter a fee of half-a-crown must be enclosed with the note containing the inquiry.**

*Tallerman Treatment.*

In reference to the query of *S. M.* in last week's issue, with the above heading, the address of the Tallerman Institute is 50 Welbeck Street, Cavendish Square, W.

*Mission Nurse.*

(1) Will you kindly tell me if there are any mission hospitals or dispensaries which may be glad of the services of a nurse for some hours daily? Remuneration not so much an object as light work in which a nurse might be useful.—*Mission Nurse.*

You will find a list of women's settlements in the "English-woman's Year Book." These are often very glad of aid.

*Incurable.*

(2) Can you tell me how to get a poor woman, suffering from an incurable spinal complaint, into an institution? She is entirely dependent on others for her support.—*F. C.*

This appears a case for the workhouse infirmary. For list of institutions receiving incurables, see "Burdett's Hospitals and Charities."

*Tuberculosis.*

(3) Should a patient, suffering from tuberculosis, be permitted to retain beard and moustache? I have heard that the germs collect about the mouth.—*Constant Reader.*

If pulmonary tuberculosis, we think he should be advised to shave.

*Long Service.*

(4) I have a friend who has been a nurse in one family for forty years. Is she not eligible for a medal for long service? I do not know how to find out, but perhaps one of your many readers may be able to tell me.—*B. L.*

Prizes and medals are offered by some domestic societies to their own members for long and continuous service, but, as far as we know, there are no prizes offered to servants, apart from these organisations.

*Canadian Trained.*

(5) Will the certificate of a nurse having had three years' training in Canada be of any use in this country?—*E. C.*

It depends upon the school bestowing the certificate whether it will be recognised as a sufficient qualification for membership by the leading nursing associations.

*Tropical Diseases.*

(6) Will you kindly give me the address of a hospital for tropical diseases which would admit a young man? He has just returned from India and is now suffering from a tropical disease.—*B. A.*

You had better apply to the Superintendent of the London School of Tropical Medicine, Seamen's Hospital Society, "Dreadnought," Greenwich, S.E.

*Insanitary Conditions.*

(7) I am with a sick sister. My sister's husband and five children sleep in the house, and there is no w.c. nor ground where refuse could be buried. Should I write to the Medical Officer of Health for the district, or to the Inspector of Nuisances?—*J. G. T.*

Write to the Inspector of Nuisances.

*Probationer.*

(8) Can you tell me if it is usual to write to the matron of any hospital asking when there would be a vacancy for a probationer? If so will you give me the address of one or two within reasonable distance of Chorley.—*Miss F. H.*

Consult "The Nursing Profession: How and Where to Train." Then apply to the matron of the training schools that appear likely to suit, asking for forms of application, and follow instructions.

I am companion-nurse to an invalid, and am making arrangements to be trained. Would you kindly tell me if the training in Kilmarnock is equal to that of King's College Hospital, London.—*M. R.*

King's College Hospital ranks as one of the first training schools in London.

1. Will you kindly tell me if having to wear spectacles will prevent my becoming a hospital nurse? 2. Can I possibly begin to train at eighteen? 3. What are the duties allotted to probationers in infirmaries?—*Bermakit.*

1. Not necessarily so. 2. In a very few children's hospitals. 3. You would find much useful information in "How to Become a Nurse" by Honnor Morton on this and other nursing topics.

1. Will you oblige me by telling me which hospitals in Yorkshire, besides Leeds and Bradford, are considered good training schools? 2. Is a Royal infirmary superior to any other?—*H. J.*

1. See answer to Miss F. H. 2. Certainly not.

1. Can you tell me of a children's or general hospital where probationers are trained with salary from the first? 2. Would training in a children's hospital, with subsequent fever training (adult), qualify nurse for Army work?—*Gerda.*

1. See answer to Miss F. H. 2. No.

*R. N. P. F. N.*

(9) Will you kindly tell me how to join the Royal National Pension Fund for Nurses?—*E. T.*

Apply to the Secretary, 28 Finsbury Pavement, E.C.

*Sanitary Inspector.*

(10) I am a trained nurse and want a lady sanitary inspector's certificate. Is it possible to get such instruction by correspondence?—*Sanitary.*

You can be prepared to a certain extent by correspondence (see our advertisement columns for a teacher), but you must have practical instruction as well. Apply for full particulars to the Secretary, The Sanitary Institute, Margaret Street, London, W.

*Home.*

(11) Can you tell me of a home for a working man's widow, aged 75? I am willing to pay 10s. a week for her.—*W. H.*

St. Cyprian's Home for the Aged Poor, 10 Little Park Street, Dorset Square, N.W. Apply Sister in Charge. Or Homes for the Aged Poor. Apply Misses Harrison, 86 Penge Road, Anerley, S.E.

Can you tell me of a home where a lady, aged 42, suffering from mental delusions, can be received? She can pay 10s. a week.—*Masseuse.*

You might write to the secretary of the Metropolitan Asylums Board, Victoria Embankment, E.C., and to the medical superintendent of your County Asylum.

*Midwifery.*

(12) I am a monthly nurse and wish to prepare for the L.O.S. examination. Which are the best and most necessary books to study.—*M. H.*

"A Practical Handook of Midwifery," by Francis W. N. Haultain, M.D., is highly recommended. By the regulations of the L. O. Society, candidates must give proof of having attended a "course of theoretical teaching by lectures or tutorial instruction." Therefore you must have a teacher as well as the book.

Having obtained my L.O.S. certificate, can you tell me of a place where I can do nothing else but midwifery?—*A. B.*

Advertisements frequently appear in our columns for midwifery nurses.

Can I join any nursing association? I have only the L.O.S. qualification.—*Nurse Alice.*

The L.O.S. does not make a person a nurse.

*Massage.*

(13) I intend taking up massage as a speciality; can you tell me where is the best place to train, what are the fees, and how long it will take me?—*F. L. M.*

Massage is thoroughly taught at the Hospital for the Paralysed and Epileptic, Queen's Square, Bloomsbury, W.C., and by others. Apply for fees to the Secretary, or to the Society of Trained Masseuses, 24 Prince's Street, Cavendish Square, W., for advice. The time taken to acquire the art depends upon natural aptitude, and upon previous knowledge.

1. Can you tell me where I can learn massage? 2. How many lessons does one usually require?—*Palæolithic.*

See reply *F. L. M.*

*India.*

(14) Will you kindly tell me what are the prospects of private nursing in India?—*B. S.*

The Up-Country Nursing Association for Europeans in India offers, after paying travelling expenses and an allowance for outfit of £20, Rs.75 a month with board, lodging, and attendance. The Hon. Sec., Major-Gen. J. Bonus, The Cedars, Strawberry Hill, W., could give you details. It would not be advisable for you to start private nursing on your own account without practical knowledge of its requirements.

Will you kindly state how I can obtain information of private nursing in India? I have heard there is an "Up-Country Nursing Association," but I have been unsuccessful in obtaining particular.—*A. C. H.*

See reply to *B. S.*

Kindly give me the address of the office of the Up-Country Nursing Association for Europeans in India?—*Eager One.*

See reply to *B. S.*

**Standard Books of Reference.**

"The Nursing Profession: How and Where to Train." 2s. net; post free 2s. 4d.

"Burdett's Official Nursing Directory." 3s. net; post free, 3s. 4d.

"Burdett's Hospitals and Charities." 5s.

"The Nurses' Dictionary of Medical Terms." 2s.

"Burdett's Series of Nursing Text-Books." 1s. each.

"A Handbook for Nurses." (Illustrated). 5s.

"Nursing: Its Theory and Practice." New Edition. 3s. 6d.

"Helps in Sickness and to Health." Fifteenth Thousand. 5s.

"The Physiological Feeding of Infants." 1s.

"The Physiological Nursery Chart." 1s.; post free, 1s. 3d.

"Hospital Expenditure: The Commissariat." 2s. 6d.

All these are published by the Scientific Press, Ltd., and may be obtained through any bookseller or direct from the publishers, 28 and 29 Southampton Street, London, W.C.

## Figures and Tables

**Fig. 1. f1:**